# Role of toll-like receptors in pulmonary immunity: mechanisms and therapeutic implications

**DOI:** 10.3389/fimmu.2025.1682649

**Published:** 2025-11-10

**Authors:** Zhuojian Qu, Zhiliang Guo, Chunjuan Yang, Xiumei Guan, Min Cheng, Pingping Wang, Donghua Xu

**Affiliations:** 1 School of Basic Medicine, Shandong Second Medical University, Weifang, China; 2 Department of Spine Surgery, The 80th Group Army Hospital of Chinese People’s Liberation Army (PLA), Weifang, China; 3 Medical Research Center, Weifang People's Hospital, Shandong Second Medical University, Weifang, China; 4 Department of Gynecology and Obstetrics, Maternal and Child Health Hospital of Shandong Second Medical University, Weifang, China; 5 Department of Rheumatology, Weifang People's Hospital, Shandong Second Medical University, Weifang, China

**Keywords:** toll-like receptor, pulmonary immunity, pattern recognition receptor, infections, homeostasis

## Abstract

Toll-like receptors (TLRs) belong to the family of pattern recognition receptors (PRRs), playing critical roles in linking innate with adaptive immunity by recognizing pathogen-associated molecular patterns (PAMPs) and danger-associated molecular patterns (DAMPs). TLRs and TLR signaling pathways serve as not only the first line of pulmonary defense against pathogens infection but crucial factors in maintaining pulmonary immune homeostasis. However, aberrant activation of TLR signaling leads to inflammation and immune dysregulations, contributing to various pulmonary diseases, including inflammation, infection, fibrosis, and malignancy. This review summarizes the updated roles of TLRs and TLR signaling in lung development and the establishment and regulation of pulmonary region-specific immunity. We further elucidate the involvement of TLRs and TLR signaling in the onset and progression of lung diseases, such as infections, fibrosis, malignancies, and immune disorders. It would provide updated insights into the exploration of novel diagnostic and therapeutic strategies targeting TLRs and TLR signaling in pulmonary diseases.

## 1 Introduction

Toll-like receptors (TLRs) belong to the family of pattern recognition receptors (PRRs) that primarily recognize pathogen-associated molecular patterns (PAMPs) and danger-associated molecular patterns (DAMPs) and activate innate immune response. They play pivotal roles in immune defense, inflammatory response, and the linkage of innate immunity with adaptive immunity. This receptor family was named due to its structural similarity to the Drosophila “Toll” protein firstly identified by Eric Wieschaus and Christiane Nüsslein-Volhard during Drosophila developmental research (1). Subsequently, TLRs have been found to be closely associated with inflammatory and immune responses (2, 3). The murine genome encodes a total of 12 functional *Tlrs*, comprising *Tlr1* through *Tlr9* along with *Tlr11* to *Tlr13*. Notably, the expression of functional *Tlr10* is absent in mice due to the insertion of retroviral-derived DNA sequences that disrupt its coding region (4). Among humans, ten functional TLRs have been identified, designated as *TLR1* through *TLR10 (*4). Based on their distinct subcellular localization patterns, TLRs can be categorized into two principal subfamilies including the cell surface subfamily and the endosomal subfamily (5). The cell surface subfamily, comprising TLR1, TLR2, TLR4, TLR5, and TLR6, is primarily localized to the plasma membrane, where these receptors recognize lipids, lipoproteins, and other extracellular PAMPs. In contrast, the endosomal subfamily, which includes TLR3, TLR7, TLR8, and TLR9, predominantly resides within intracellular compartments, such as the endoplasmic reticulum, endosomes, and lysosomes, where they mediate the detection of nucleic acids derived from intracellular pathogens (5).

From a molecular perspective, TLRs are type I single-pass transmembrane proteins, ranging from 700 to 1, 100 amino acids in length, whose extracellular leucine-rich repeat (LRR) domains serve as sensors for PAMPs, thereby triggering the activation of innate immunity (6, 7). The intracellular Toll/IL-1 receptor (TIR) domain is evolutionarily conserved and serves as a signaling platform for the recruitment of specific adaptor proteins, such as TIR domain-containing adaptor protein (TIRAP), myeloid differentiation factor 88 (MyD88), TIR-domain-containing adaptor inducing interferon-β (TRIF), and TRIF-related adapter molecule (TRAM). This assembly nucleates distinct signaling complexes that activate nuclear factor-κB (NF-κB) and interferon regulatory factor (IRF) transcription factors, leading to the production of proinflammatory cytokines and type I interferons (IFNs) (8). With the exception of TLR3 and the endosomal TLRs (TLR7/8/9), select TLRs (TLR2 and TLR4) require the bridging adaptor TIRAP to recruit MyD88, which in turn activates interleukin-1 receptor-associated kinases (IRAKs) and downstream NF-κB/Mitogen-Activated Protein Kinase (MAPK) pathways to induce proinflammatory cytokines (9). In contrast, TLR3 signals independently of MyD88 by engaging the adaptor TRIF, which activates TANK-binding kinase 1 (TBK1) and IκB kinase ϵ (IKKϵ) to phosphorylate IRF3, thereby inducing IFNs and contributing to delayed NF-κB activation (10, 11). TLR4 is unique in its ability to utilize both the MyD88-TIRAP and TRIF-TRAM axes, enabling it to orchestrate robust inflammatory responses alongside potent antiviral interferon production (12). Furthermore, endosomal TLRs, such as TLR7, TLR8, and TLR9, recognize nucleic acid ligands and can directly engage MyD88 to recruit and activate IRF7, driving rapid and robust type I interferon responses (13). Collectively, these specialized signaling architectures enable precise control of immune cell activation and effector functions, playing pivotal roles in establishing pulmonary immunity and shaping the pathogenesis of lung diseases.

TLRs are expressed in various types of immune cells, including macrophages, dendritic cells (DCs), B lymphocytes, and T lymphocytes (14). They regulate the expression of pro-inflammatory cytokines and interferons by activating key transcription factors, such as NF-κB and IRFs, thereby aiding the host in defending against a wide range of pathogenic infections and adapting to complex microenvironmental changes (15). In the lung, TLRs are predominantly expressed in immune cells such as alveolar macrophages, DCs, and lymphocytes, forming the foundation of both innate and adaptive immune responses in the respiratory system (16). Studies have shown that TLRs play critical roles in the initiation and progression of lung diseases. For instance, activation of mucosal TLR5 has been demonstrated to delay thymic involution and protect against pulmonary fibrosis through enhancement of stem cell activity (17). X-linked recessive *TLR7* deficiency in males results in impaired IFN immunity and severe COVID-19 pneumonia (18). In a house dust mite-induced murine model of allergic asthma, activation of TLR3 not only enhanced the antiviral response but alleviated the viral infection *via* regulating immunoproteasome dysfunction (19). In addition to immune cells, TLRs are also expressed in pulmonary epithelial cells and vascular endothelial cells, which play regulatory roles in maintaining lung function (20). Therefore, TLRs are essential for defending against pulmonary infections and maintaining regional immunity balance. However, excessive activation of TLRs can lead to pulmonary inflammation and immune dysregulation, contributing to the development of pneumonia, pulmonary fibrosis, and lung cancer. It has been shown that TLR4-mediated chronic inflammatory responses lead to an imbalance in the proportions of alveolar macrophages and CD163^+^ myeloid-derived monocyte-macrophages, which represents one of the fatal mechanisms underlying COVID-19 pathogenesis (21). Air pollutants such as polystyrene microplastics can induce pulmonary inflammation and apoptosis of lung cells by activating the TLR2/NF-κB signaling pathway, ultimately leading to lung injury and fibrosis (22). Therefore, the TLR family plays a crucial role in the regulation of pulmonary inflammation and regional immunity, representing a potential therapeutic target for the intervention of lung diseases.

In this review, we aim to elucidate the regulatory roles and underlying mechanisms of TLRs in lung physiology, as well as the immunomodulatory functions of TLRs and their downstream signaling molecules in pulmonary immunity. Furthermore, we discuss how aberrant activation of TLR signaling contributes to the pathogenesis of various lung diseases, including pulmonary infectious diseases, interstitial lung diseases (ILDs), and malignancies. We also briefly summarize recent clinical studies targeting TLR pathways, highlighting their potential for therapeutic intervention. This work provides a theoretical foundation for the development of novel strategies targeting TLRs and their signaling networks in the treatment of pulmonary disorders.

## 2 Regulatory roles of TLRs in pulmonary physiology

### 2.1 TLRs in maintaining pulmonary homeostasis

As one of the first identified PRRs, TLRs play a pivotal role in the regulation of innate immunity by recognizing PAMPs and DAMPs (23). In the lung, TLRs are expressed not only in immune cells, such as alveolar macrophages and dendritic cells, but also in pulmonary epithelial cells, suggesting their critical roles in host defense against infection and normal lung development (24, 25). Using a false discovery rate algorithm, researchers have found that TLR2 was consistently upregulated across distinct stages of fetal lung development, from the early pseudo-glandular stage to the late pseudo-glandular and canalicular phases (25). In addition, the functional expression of TLR2 and TLR4 has been detected in murine pulmonary epithelial cells (26). Upon recognition of pathogen-derived molecules, these receptors promote epithelial cell proliferation (27). Studies utilizing gene knockout technology have demonstrated that *Tlr2*^-/-^ and *Tlr4*^-/-^ mice exhibit enhanced pulmonary epithelial cell apoptosis and impaired macrophage trans-epithelial migration following lung injury (28). These findings suggest that TLR2 and TLR4 play critical roles in maintaining epithelial cell integrity and facilitating tissue repair following lung injury. The protective effects of TLR2 and TLR4 on epithelial cells are predominantly mediated through the recognition of intracellular high-molecular-weight hyaluronic acid (HA) (29). As a critical mediator of tissue repair and remodeling, hyaluronic acid not only inhibits cellular apoptosis but promotes the proliferation and regeneration of surfactant protein C-positive alveolar progenitor cells through TLR4 activation, thereby inhibiting pulmonary fibrosis in mice (30, 31).

**Figure 1:**
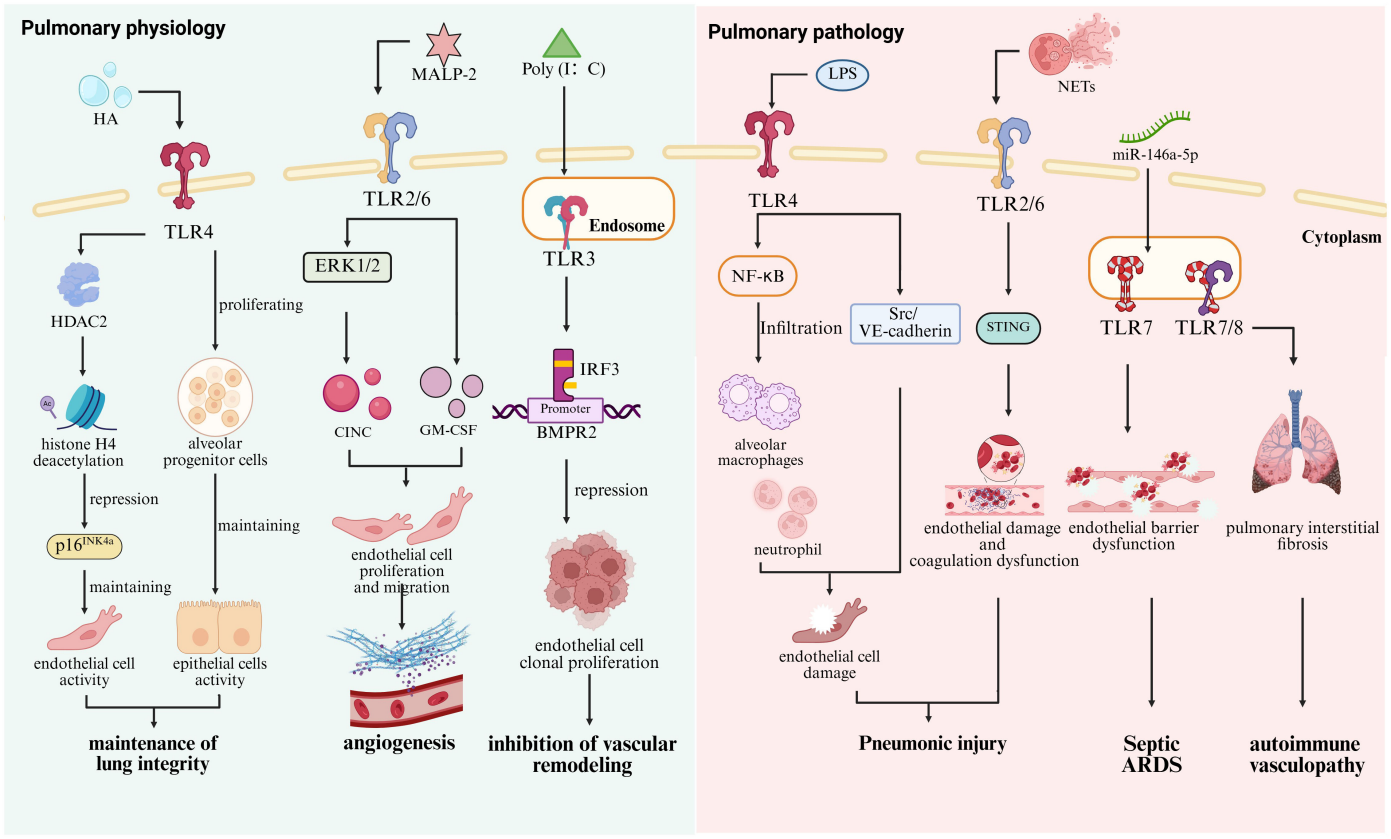
Roles of TLRs in pulmonary physiology and pathology. In lung physiological homeostasis, TLR4 senses intracellular HA to promote the proliferation and renewal of alveolar progenitor cells, while its activation in endothelial cells induces histone H4 deacetylation via HDAC2-mediated mechanisms, leading to the suppression of the senescence-associated gene p16INK4a and the maintenance of pulmonary integrity (30, 31, 37). The TLR2/6 agonist MALP-2 mediates ERK1/2 phosphorylation and CINC secretion upregulates GM-CSF expression and promotes pulmonary angiogenesis (34). In contrast, the TLR3 agonist Poly (I: C) suppresses endothelial cell clonogenic proliferation and attenuates vascular remodeling by enhancing IRF3 binding to the BMPR2 promoter (36). In contrast, excessive activation of TLR4 triggers Scr/VE-cadherin pathway activation and promotes alveolar macrophages and neutrophils through NF-κB hyperactivation (132). TLR2 in endothelial cells exacerbates endothelial injury and coagulation dysregulation by mediating NETs-STING interactions (130). In addition, TLR7 recognizes miR-146a-5p, leading to impaired endothelial barrier function and contributing to the development of sepsis-induced ARDS (133), while excessive TLR7/8 activation drives autoimmune vasculitis (134).

Endothelial cells are essential cells maintaining the pulmonary homeostasis through the expression of various adhesion molecules and cytokines (32). Studies have shown that *Tlr2* deletion in murine pulmonary endothelial cells leads to a significant reduction in angiogenesis-associated signaling pathways, including the phosphorylation activation of extracellular signal-regulated kinases 1 and 2 (ERK1/2), as well as the secretion of cytokine-induced neutrophil chemoattractant (CINC) (33). As a TLR2/6 agonist, macrophage-activating lipopeptide 2 kDa (MALP-2) not only promotes the proliferation and migration of endothelial cells but upregulates the expression of granulocyte-macrophage colony-stimulating factor (GM-CSF) essential for angiogenesis (34). Emerging evidence indicates that the expression of TLR3 is significantly downregulated in pulmonary endothelial cells from patients with pulmonary arterial hypertension (PAH). Knockout of *Tlr3* enhances the susceptibility of endothelial cells to apoptosis in *Tlr3*-deficient (*Tlr3*^−/−^) mice, thereby contributing to pulmonary vascular remodeling (35). Furthermore, the TLR3 agonist polyinosinic/polycytidylic acid [Poly(I: C)] enhances the binding of IRF3 to the bone morphogenetic protein receptor II (BMPR2) promoter, thereby inhibiting clonal proliferation of endothelial cells and alleviating pulmonary arterial hypertension (PAH) caused by vascular remodeling (36). Activation of TLR4 suppresses the expression of p16^INK4a^, a senescence-associated protein, *via* histone deacetylase 2 (HDAC2)-mediated deacetylation of histone H4 (37). However, the silencing of *Tlr4* in pulmonary endothelial cells leads to the development of emphysema. Accordingly, TLRs contribute to the maintenance of pulmonary integrity by regulating endothelial cells (**Figure 1**).

### 2.2 TLRs and pulmonary microbiome

The lung microbiota is closely associated with the maintenance of pulmonary homeostasis and the regulation of local alveolar immune responses, while the pulmonary immunity is crucial for the maintenance of lung Microbiome (38). In *Tlr*-deficient mice, the pulmonary microbiota exhibits significant dysbiosis, indicating that TLRs play a crucial role in regulating lung microbiome (39). However, selective activation of TLRs does not alter the gut microbiota in healthy mice, suggesting that under normal physiological conditions, TLR signaling has limited influence on microbial community composition (40). It has been shown that the expression of TLR9 in the lung is positively correlated with the abundance of *Staphylococcus* and *Prevotella*, and the interaction between TLR9 and the microbiota is associated with improved progression-free survival (PFS) in pulmonary fibrosis (41). These findings suggest a potential role for TLR9 in modulating the pulmonary microbiota and its impact on the pathogenesis of pulmonary fibrosis. Besides, the responsiveness of TLR4 in alveolar macrophages is reduced in individuals with a pneumotype characterized by enrichment in upper respiratory tract-associated microbiota (pneumotype SPT), and this reduction is associated with attenuated pulmonary inflammatory response (42). This difference reflects the distinct regulatory mechanisms by which different pulmonary microbiota modulate immune responses in the lung. These findings indicate that the activation of TLRs not only directly influences the composition of the pulmonary microbiota, but indirectly affects microbiota dynamics by modulating pulmonary immune and inflammatory responses.

In summary, TLRs play an indispensable role in lung development and physiological regulation. They contribute to the maintenance of normal pulmonary function through the modulation of lung epithelial and vascular endothelial cells, as well as the complicated interactions with the pulmonary microbiota. TLRs not only contribute to the maintenance of pulmonary homeostasis, but serve as key foundation linking both innate and adaptive immune defenses. Moreover, TLRs and the subsequent activation of downstream signaling pathways can trigger a range of pathophysiological changes in the lung. The functional heterogeneity of TLRs provides insight into understanding the mechanistic roles of TLRs in various pulmonary diseases.

## 3 Orchestrating immunity and inflammation: functions of TLR adaptors in the lung

TLRs play a pivotal role in lung development and homeostasis through recognition of specific ligands, a process reliant on highly conserved downstream signaling pathways and specialized adaptor molecules. Key adaptors, including MyD88, TIRAP, TRIF, and TRAM, form a core signaling network that not only provides a first line of defense against pathogens but also ensures immune homeostasis and prevents excessive inflammation (**Table 1**).

**Table 1:** Functions of TLR adaptor proteins in lung immunity.

Adaptor molecules	Related TLRs	Downstream signaling pathway	Functions in lung immunity	Refs.
MyD88	TLR2, TLR4, TLR5, TLR7/8, TLR9	1.MyD88- IRAK1/4- MAPK /NF-κB 2. MyD88 - IRAK1 - IRF7	Protective Roles: •Clearing bacterial infections and inducing proinflammatory cytokines (TNF-α, IL-6)	(43)
			•Mounting antiviral defense via IRF7-mediated IFN-α induction	(13)
			•Synergizing with cGAS-STING to boost IFNγ production in Ly6Chi monocytes against pneumococcus	(46)
			Pathogenic Roles: •Exacerbating ALI by driving macrophage overactivation	(50)
TIRAP	TLR2, TLR4	TIRAP - MyD88 - NF-κB	Protective Roles: •Serving as a dedicated adaptor for recruiting MyD88 to membrane-bound TLR2/4	(47)
			•Mediating Antiviral and Antibacterial Responses in the Lung	(48, 49)
TRIF	TLR3, TLR4	TRIF - TBK1/IKKϵ - IRF3	Protective Roles: •Mediating antiviral responses through IFN-β production	(55)
			• Contributing to OM-85-induced Treg expansion and suppression of type 2 asthma inflammation	(56)
			Pathogenic Roles: •Mediating tissue damage via caspase-8/GSDMD pyroptosis pathway during chronic inflammation	(57)
TRAM	TLR4	TRAM - TRIF - TBK1/IKKϵ - IRF3	Protective Roles: •Guiding TLR4-TRIF pathway activation for antiviral immunity	(55)
			•Interacting with NLRC3 in Tregs to suppress excessive inflammation and pathological vascular remodeling	(60)
			Pathogenic Roles: •Converting neutrophils to a pro-inflammatory phenotype, exacerbating lung injury in experimental sepsis.	(59)

### 3.1 MyD88 and TIRAP

As the central adaptor for most TLRs, MyD88 recruits IRAK1 and IRAK4 *via* its death domain to form the Myddosome complex, activating the MAPK and NF-κB signaling pathways. This leads to the nuclear translocation of NF-κB and AP-1, rapidly inducing the pro-inflammatory cytokines such as TNF-α and IL-6, which are essential for bacterial clearance in the lung (9, 43). Within endosomes, MyD88 is recruited by TLR7, TLR8, and TLR9 to initiate the MyD88-IRF7 signaling axis, driving the phosphorylation and nuclear translocation of IRF7, resulting in robust production of IFN-α critical for antiviral immunity (13).

Studies have shown that *MyD88*-deficient mice exhibit significantly higher viral loads in the lungs following SARS-CoV infection (44), and display increased susceptibility and mortality during *Streptococcus pneumoniae* infection (45). These findings underscore the critical role of MyD88 in pulmonary host defense against both viral and bacterial pathogens. MyD88 synergizes with the cyclic GMP-AMP synthase-stimulator of interferon genes (cGAS-STING)pathway in Ly6C^hi^ monocytes to enhance IFN-γ production during *Streptococcus pneumoniae* infection (46). TIRAP facilitates MyD88 recruitment to TLR2/4 complexes (47). Similarly, *Tirap*-deficient mice exhibit increased mortality in bacterial lung infections. Studies have demonstrated that TIRAP is a critical mediator in the lung's defense against *Klebsiella pneumoniae* and *Escherichia coli* infections (48, 49).

However, during SARS-CoV-2 infection, aberrant activation of the MyD88/TIRAP-IRAK-NF-κB signaling axis may drive macrophage hyperactivation and cytokine storm-mediated acute lung injury (ALI) (50). Targeting this axis has emerged as a therapeutic strategy (51, 52). Interestingly, TIRAP-MyD88 inhibition promotes M2 macrophage polarization, underscoring its context-dependent role (53). MyD88 function also varies by cell type: in myeloid cells it exacerbates inflammation, whereas in stromal cells it may exert anti-inflammatory effects (54).

### 3.2 TRIF and TRAM

TRIF is encoded by the *Ticam1* gene. The TRIF-dependent pathway, activated primarily by TLR3/4, induces IFN-β production. TRIF recruits TBK1 and IKKϵ, leading to IRF3 phosphorylation, nuclear translocation, and IFNB1 transcription. *Ticam1* deficiency impairs this antiviral response (55). TRAM specifically bridges TLR4 to TRIF; its deletion disrupts TLR4-mediated TRIF-TBK1-IRF3 activation and increases viral susceptibility (55).

Beyond antiviral roles, TRIF signaling has immunomodulatory functions. The bacterial lysate OM-85 expands Tregs *via* dendritic cell MyD88/TRIF signaling, suppressing type 2 inflammation in asthma and promoting tolerance (56). However, in chronic inflammation such as cigarette smoke exposure, TLR4 signaling may shift from MyD88 to TRIF/caspase-8/GSDMD pyroptosis, releasing DAMPs and perpetuating tissue injury (57). Thus, MyD88 and TRIF are not simply antagonistic but form a dynamic network with bidirectional crosstalk, where outcomes depend on stimulus, cell type, and microenvironment. For instance, in ALI, TLR4 synergistically activates STING *via* coordinated MyD88 and TRIF signaling, amplifying inflammation (58).

Targeting TLR adaptors offers novel therapeutic potential. In experimental sepsis, TRAM deletion promotes neutrophil resolution and reprograms monocyte/macrophage function, alleviating lung injury (59). TRAM also interacts with NLRC3 in Tregs to suppress excessive inflammation and pathologic vascular remodeling (60).

Despite advances, key challenges remain. Cell type-specific functions of adaptors are incompletely defined. In chronic diseases, precise modulation, such as inhibiting detrimental MyD88-driven inflammation while preserving beneficial TRIF-mediated responses, remains a major hurdle. Studies on downstream adaptor proteins of TLRs in the lung have revealed that these adaptors are essential mediators of TLR-mediated immune defense and immunoregulation. Dysregulation of adaptor function can lead to excessive TLR activation and contribute to the development of pulmonary pathological changes. In the following sections, we will discuss the roles of TLRs in regulating both innate and adaptive immunity in the lung, as well as the mechanisms by which dysregulation of the TLR signaling network drives pulmonary disease pathogenesis.

## 4 Regulatory network of TLRs in pulmonary regional immunity

### 4.1 Regulation of innate immune responses by TLRs in the lung

The innate immune system in the lungs constitutes the first line of defense against pathogen invasion through rapid response mediated by PRRs (61). Among PRRs, TLRs initiate innate immune response upon recognition of PAMPs. Innate immune cells such as alveolar macrophages, dendritic cells, and neutrophils establish a defense network within the pulmonary microenvironment *via* TLRs signaling pathway (62).

#### 4.1.1 Regulation of alveolar macrophages by TLRs

**Figure 2:**
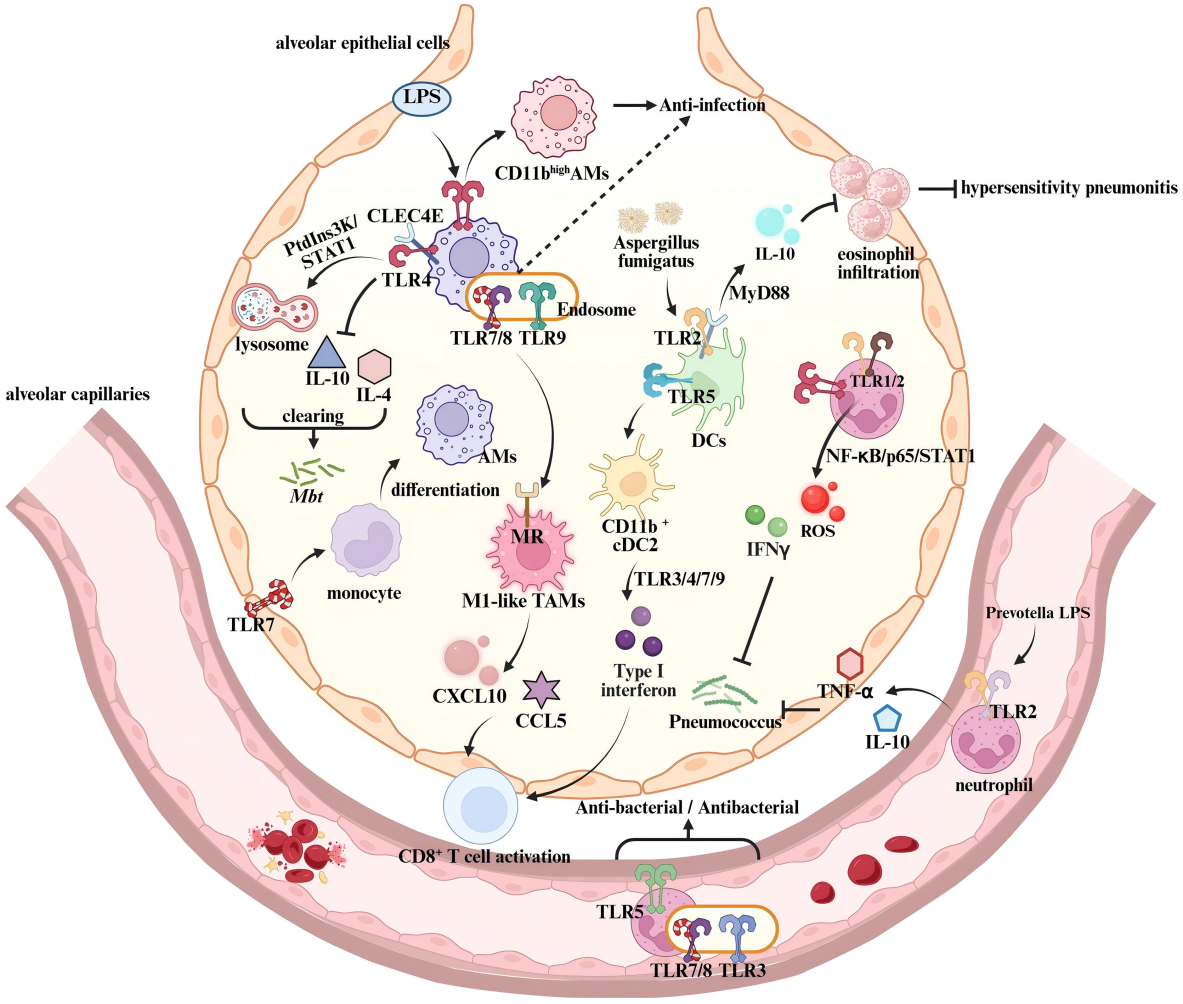
Regulation of innate immune responses by TLRs in the lung. In Alveolar macrophages, the co-activation of TLR4 and CLEC4E triggers the MyD88/PtdIns3K/STAT1/NF-κB signaling pathway, enhancing lysosome biogenesis while suppressing IL-10 and IL-4 expression, thereby controlling Mtb infection (68). LPS-activated TLR4 induces a phenotypic transition from CD11blow to CD11bhigh Alveolar macrophages, modulating their response to pathogen-associated components (72). TLR7 activation in epithelial barriers promotes monocyte differentiation into AMs, reducing pulmonary viral load (74). In the tumor microenvironment, endosomal TLR7/8 activation synergizes with MR signaling to drive TAMs toward an M1 anti-tumor phenotype (82). This enhances T cell recruitment by upregulating chemokines CXCL10 and CCL5, thereby boosting anti-tumor immunity. During Aspergillus fumigatus infection, TLR2-CLEC4E co-activation in dendritic cells increases IL-10 production via MyD88, suppressing eosinophil infiltration and negatively regulating hypersensitivity pneumonitis (69). TLR5 signaling promotes dendritic cell differentiation into CD11b^+^ cDC2 subset, which then releases type I interferons through TLR3/4/7/9 activation, enhancing T cell function (86, 87). Neutrophil-expressed TLR2 recognizes lipoproteins from Prevotella species in airways, inducing TNF-α and IL-10 production (75). TLR1/2 and TLR4 activation triggers NF-κB/p65/STAT1 signaling, promoting ROS and IFN-γ release, which mediate pulmonary antibacterial immunity (91, 92).

As specialized tissue-resident macrophages localized within the alveolar lumen and interstitium, alveolar macrophages play a central role in respiratory immune defense through unique tissue adaptability and phenotypic plasticity (63). Activation of TLRs is not only essential for the initiation of phagocytic function in alveolar macrophages but also facilitates the formation of immunorecognition complex through synergistic interactions with other PRRs (**Figure 2**) (64). It has been well demonstrated that TLR2 recognizes the influenza virus and mediates the establishment of an antiviral defense barrier in the upper respiratory tract, thereby significantly reducing the risk of viral dissemination to the pulmonary parenchyma (65). In a *Mycobacterium tuberculosis* (Mtb) infection model, the activation of TLR2/Radioprotective 105 kDa protein (RP105) signaling axis in alveolar macrophages promotes the expansion of the macrophage-rich region at the granuloma core (66). TLR4 forms a heterodimeric complex with the C-type lectin receptor CLEC4E, enhancing lysosome biogenesis through the phosphoinositide 3-kinase(PI3K)-STAT1 signaling pathway while simultaneously suppressing the secretion of Th2-type cytokines, such as IL-4 and IL-10, thereby enabling the clearance of Mtb (67, 68). This synergistic effect is receptor-specific. In allergic pneumonia caused by *Aspergillus fumigatus*, the co-activation of TLR2 and CLEC4E in bone marrow-derived dendritic cells suppresses inflammation *via* upregulating IL-10 in a MyD88-dependent manner (69).

The functional diversity of TLRs is particularly evident in bacterial pneumonia. During *Streptococcus pneumoniae* infection, the deficiency of endosomal TLR-mediated (TLR7/9) nucleic acid sensing pathways in alveolar macrophages leads to enhanced infection. Notably, a functional compensation between TLR7 and TLR9 in nucleic acid recognition has been observed, which plays a role in preventing *S. pneumoniae* from immune evasion (70). In addition, alveolar macrophages undergo phenotypic transition upon TLR activation, which affects the production of pro-inflammatory cytokines and chemokines (71). For example, in a lipopolysaccharide (LPS)-induced murine model of acute respiratory distress syndrome (ARDS), alveolar macrophages undergo a TLR4-mediated phenotypic transition from CD11b^low^ to CD11b^high^, thereby enhancing the inflammatory response to pathogens (72). These findings confirm the central role of TLRs in the regulation of alveolar macrophages and highlight their contributions to enhanced pulmonary immune responses through synergistic interactions with other PRRs.

The immunoregulatory network of TLRs is also essential for the remodeling of tissue microenvironment. In *Legionella pneumonia*, infected alveolar macrophages induce an interleukin-1 (IL-1)-dependent inflammatory response, which thus stimulates alveolar epithelial cells to produce GM-CSF (73). GM-CSF signaling enhances TLR-mediated pathways in alveolar macrophages, leading to metabolic reprogramming characterized by increased glycolysis, thereby amplifying the antimicrobial activity and inflammatory cytokine production of monocytes (73). Moreover, the activation of TLR7 promotes the differentiation of pulmonary monocytes into tissue macrophages, significantly reducing pulmonary viral load (74). Neutrophil-expressed TLR2 plays a crucial role in the clearance of *S. pneumoniae* by recognizing lipoproteins of *Prevotella* species and enhancing serine protease activity (75). TLR2 agonist INNA-X activates the TLR2/NF-κB/IFN-λ signaling pathway in airway epithelial cells, thereby enhancing lymphocyte recruitment and suppressing neutrophils-mediated inflammation (76). Accordingly, TLRs help to establish a sustained innate immune response that alleviates pulmonary infections.

Alveolar macrophages exert immunosuppressive effects during the anti-tumor immune response (77). Emerging evidence indicates that TLRs enhance the efficacy of cancer immunotherapy by modulating metabolic reprogramming in alveolar macrophages (78). It has been demonstrated that HA-mannose-modified nanocapsules loaded with TLR3 agonist Poly (I: C) and TLR7/8 agonist resiquimod (R848) could specifically target alveolar macrophages in lung tumor-bearing mice (79). Activation of TLR3/7/8 induces alveolar macrophages to an CD86^high^CD206^low^Arg1^low^ M1-like antitumor phenotype, enhancing the expression of T-cell chemokines CXCL10 and CCL5 and effectively suppressing tumor metastasis (79). SHISA3 functions as a tumor-suppressive protein (80). The combination of the TLR4 agonist monophosphoryl lipid A (MPLA) and anti-PD-1 antibody promotes SHISA3 expression *via* the NF-κB pathway, thereby promoting antitumor M1 polarization and phagocytic capacity of alveolar macrophages (81). In addition, the TLR7/8 agonist imiquimod (IMDQ) conjugated to nanobodies regulates the mannose receptor (MR) and induces M1-like repolarization of alveolar macrophages, which obviously suppresses tumor progression (82). Taken together, these findings highlight the critical role of TLRs in regulating phenotypic transitions of alveolar macrophages during the antitumor immunity. Targeting TLRs in alveolar macrophages using agonists holds promise as a novel therapeutic strategy for pulmonary cancer.

#### 4.1.2 TLRs regulate pulmonary dendritic cells

DCs are pivotal antigen-presenting cells in the immune system, serving as a bridge between innate and adaptive immunity (83). TLRs play a crucial role in modulating the phenotype and function of pulmonary DCs (**Figure 2**). For instance, TLR2 activation induces increased reactive oxygen species (ROS) production, which enhances antigen presentation and immune response in the lung (84). The TLR3 agonist Poly (I: C) activates pulmonary DCs, thereby promoting the recruitment of natural killer (NK) cells and the activation of CD8^+^ T cells (85). Besides, the TLR5 agonist flagellin promotes the expression of maturation markers such as CD40, CD80, and CD86 on lung conventional DC subsets (CD103^+^ cDC1 and CD11b^+^ cDC2), and significantly enhances their migration to mediastinal lymph nodes in neonatal mice, thereby facilitating the establishment of pulmonary mucosal immunity (86). In a murine model of respiratory infection, conventional DC type 2 (cDC2) activates TLR3/4/7/9 and downstream signaling pathways, leading to elevated type I IFNs and the inflammatory cDC2s. These inf-cDC2s exhibit a robust capacity to promote the polarization of CD4^+^Th cells toward a Th1 bias and the antigen-presenting capability to CD8^+^T cells (87).

In tumor-associated DCs, combined applications of TLR7/8 agonists and STAT3 inhibitors effectively enhance the antigen uptake and presentation by DCs, which thus promotes DC migration to lymph nodes and augments the antigen-specific cytotoxic activity of CD8^+^ T cells (88). The activation of TLR9 not only induces the expansion of tumor-associated DCs, but elicit the antitumor immune response by synergizing with PD-L1 inhibitors (89). Although studies on TLR-targeted modulation in pulmonary DCs remain limited, evidence from current research in other organs suggests that tissue-resident DCs may influence tumor development and progression by modulating the balance of the local immune microenvironment. The potential effects and mechanisms of DCs in pulmonary cancer immunity warrant further investigations in the future.

Thus, TLRs modulate the phenotype and function of DCs through distinct signaling pathways, thereby influencing T-cell activation and the magnitude of immune responses. In infection and tumor models, TLR activation significantly enhances the antigen-presenting capacity and immunomodulatory functions of DCs, providing new insight into the exploration of immunotherapy approaches in pulmonary cancer.

#### 4.1.3 TLRs modulate lung neutrophils function

Neutrophils are critical effector cells involved in pulmonary innate immune response. Upon pathogen invasion, neutrophils rapidly migrate to the site of infection and recognize PAMPs *via* TLRs (90) (**Figure 2**). In a mouse model of *S. pneumoniae*-induced pneumonia, the activation of TLR1/2 and TLR4 and TANK-binding kinase 1 phosphorylation in neutrophils through the NF-κB/p65/STAT1 signaling pathway promotes the expression of ROS, IFN-γ, and IL-12p40, mediating pulmonary antibacterial immunity (91, 92). Studies have also demonstrated that activation of TLR3 and the TLR5 both enhance the early mobilization of neutrophils and pulmonary antibacterial activity (93, 94). In tumor microenvironment (TME), pulmonary neutrophils exert both antitumor and protumor effects (95). Tumor-associated neutrophils (TANs) are a critical component of the premetastatic niche (PMN) in the lung. Activation of TLR signaling pathways promotes the recruitment of TANs and their polarization toward an N2 phenotype (pro-tumorigenic), thereby accelerating lung cancer metastasis (96). In non-small cell lung cancer (NSCLC), neutrophils are activated by Annexin A2 *via* the TLR2-MyD88 axis, leading to increased expression of arginase 1 (97). This induction results in severe dysfunction of T cells and compromises pulmonary antitumor immune responses. However, activation of TLR7/8 in pulmonary neutrophils enhances their phagocytic capacity against tumor cells, thereby effectively inhibiting the progression of lung cancer (98). These findings highlight the potential therapeutic role of TLRs and TLRs signaling pathways in regulating neutrophils in lung cancer.

In summary, TLRs serve as the core "immune sentinels" of pulmonary innate immunity *via* regulating the functions of alveolar macrophages, DCs, neutrophils, and other effector cells. TLRs play vital roles in the rapid recognition and clearance of pathogens, cascading inflammatory response, and antitumor immunity in the lung, collectively maintaining pulmonary homeostasis. Most importantly, the role of TLRs extends beyond innate immunity, serving as a bridge linking innate immunity with adaptive immunity.

### 4.2 Regulation of adaptive immunity by TLRs in the lung

#### 4.2.1 TLRs regulate pulmonary T lymphocytes

TLRs play a central role in pulmonary adaptive immunity by regulating T-cell functions (99). In an Mtb infection model, the absence of TLR2 signaling significantly impairs the co-stimulatory capacity of CD4^+^ and CD8^+^ T cells, resulting in decreased cytokines production, such as IFN-γ, TNF-α, and IL-10 (100). Notably, TLR2 plays a distinctive role in respiratory vaccine immune responses. Studies on SARS-CoV-2 mucosal vaccines have demonstrated that co-administration of the spike protein with TLR2 agonist Pam2Cys significantly increases the proportion of spike-specific T follicular helper cells, the capacity of CD4^+^ T cells to produce IL-17A and TNF, and the generation of anti-spike IgA and neutralizing antibody levels (101). In contrast to TLR2, intranasal subunit vaccines containing TLR3 agonists in cationic liposomes effectively induce airway IgA production and pulmonary CD4^+^ and CD8^+^ T cell responses (102). Besides, the adjuvant system incorporating the TLR3 agonist NexaVant more efficiently promotes the expansion of lung tissue-resident memory T cells *via* a type I IFN-dependent pathway (103). Furthermore, the combination of the MVA-SARS-2-S vaccine with a TLR3 agonist significantly increases the number of pulmonary CD8^+^ T cells (104). In contrast, the TLR9 agonist CpG primarily enhances cellular immune response by promoting pulmonary CD8^+^ cytotoxic T lymphocytes differentiation and the expression of granzyme B (105). TLR2 activation induces CD4^+^ T cells to differentiate into CD4^+^CD25^+^FOXP3^+^ Tregs, which leads to increased viral load in the *Aspergillus fumigatus* infection model (106). Similarly, in paracoccidioidomycosis (PCM), TLR3 facilitates fungal immune evasion by inhibiting the activation and cytotoxic function of IFN-γ^+^CD8^+^ T and IL-17^+^CD8^+^ T cells (107). The TLR2/4 signaling positively correlates with infection severity due to increased expression of suppressive factors, such as PD-L1, IL-10, and nitrotyrosine in myeloid-derived suppressor cells (MDSCs), which significantly impairs T cell antifungal activity (108). This suggests TLRs play critical roles in regulating T Lymphocytes during pulmonary infections.

The functional plasticity of pulmonary T cell responses is critically shaped by TLRs. For example, histone components within NETs induce TLR2 activation and STAT3 phosphorylation in T cells, thereby driving Th17 polarization (109). Similarly, during Mtb infection, TLR4-MyD88 signaling orchestrates DC maturation and cytokine production, notably IL-12p70 and IL-23p19, *via* T-bet upregulation. This process facilitates the differentiation of CD4^+^ T cells into Th1 and Th17 subsets, which are critical for effective antimicrobial immunity (110). Interestingly, TLR4 agonist glucopyranosyl lipid adjuvant suppresses the differentiation of pulmonary CD8^+^ T cells by limiting T cell receptor signaling, thereby promoting respiratory mucosal immunity *via* upregulating memory T cell formation and TH17/TC17 responses (111). In contrast, TLR9 agonist CpG promote TH1/TC1 effector cells expansion but inhibiting TH17 differentiation (111). In NSCLC, TLR3/TLR7 agonists effectively counteract TGF-β-mediated immunosuppression by inducing IFN-γ production, thereby inhibiting Treg expansion (112). Activation of NF-κB and IRF3 signaling pathways enhances CD8^+^ T cell functions, promoting antitumor immunity. Additionally, in lung adenocarcinoma models, the efficacy of antitumor drugs is closely related to the cytotoxic function of CD8^+^ T cells mediated by TLR4 (113). Treatment with a TLR9 agonist in combination with TGF-β2 inhibitor enhances the antitumor activity of CD8^+^ T cells (114). Accordingly, TLRs are essential for T cells-mediated tumor immunity in the lung. All these findings highlight the complex regulatory networks of TLRs in pulmonary adaptive immunity. Nonetheless, the specific mechanisms by which TLRs regulate T lymphocytes in the lungs still require further investigations in future studies.

#### 4.2.2 Regulation of pulmonary B lymphocytes by TLRs

TLRs regulate B cell-mediated humoral immunity in the lungs through both B cell-intrinsic signaling and microenvironment-dependent pathways. TLR4 collaborates with B cell receptor *via* the TLR4-TRIF pathway to induce the production of the monocyte chemoattractant CCL7, a molecule critical for initiating neutrophil extravasation and monocyte recruitment in the lungs (115). In a Brucella infection model, pulmonary B cell TLR2/4 is essential for the early IgG production, while downstream MYD88 activation is associated with the production of antigen-specific IgG in the later stages (116). In antiviral immunity, the TLR7-IRF7-IFNα/γ axis directly affects the efficiency of antiviral antibody production by B cells. Double-knockout of both *Tlr7* and *Irf7* leads to reduced IFN-α and IFN-γ, impaired antibody production, and delayed viral clearance in the lungs (117). Notably, the combination of TLR7 agonist imiquimod with inactivated viral particles can directly induce naïve B cells to differentiate into plasma cells, highlighting the critical role of TLR signaling in B cells response (118). Moreover, the maintenance of glycolytic metabolic activity and mitochondrial homeostasis in B cells depends on TLR9 signaling and the co-stimulation by helper T cells (119). This regulatory mechanism not only enhances the anti-apoptotic capacity of B cells, but promotes their differentiation into effector B cells. Notably, in the context of autoimmune pathology, abnormal activation of B cells by TLRs can lead to pathological responses. For instance, small nuclear RNAs can activate B cell TLR7, driving the production of anti-dsDNA and anti-Smith antibodies in SLE (120). In patients with systemic sclerosis (SSc), the intrinsic hyperactivation of TLR9 in B cells contributes to immune dysregulation (121). Aberrant activation of TLR9 in regulatory B cells (Bregs) further disrupts the function of the STAT3 and p38 MAPK signaling pathways, leading to a reduction in Breg and abnormal upregulation of CD19 (122). In a mouse model of SSc, CD19 deficiency has been shown to significantly attenuate lung fibrosis and autoantibody production in response to TLR4 activation (123). Accordingly, targeting TLRs pathway may represent a novel therapeutic strategy for autoimmune-mediated lung injury.

It has been well documented that the activation of TLR3 in lung epithelial cells leads to the release of B cell activating factor, which effectively promotes the survival of memory B cells and plasma cell differentiation (124). In contrast, excessive activation of TLR9 exerts anti-inflammatory effects in the lung by inducing Bregs to secrete IL-10 (125). Besides, the TLR7/9 signaling pathway has been shown to play a unique role in adaptive immune response by driving the IgD^+^CD21^-^CD23^-^ age-associated B cells (ABCs) differentiation into infection-induced ABCs and memory B cells, which are crucial for defending against influenza A virus infection among elderly individuals (126). Moreover, in a schistosome infection model, reduced response of lung B cells to TLR4/9 stimulation leads to decreased IL-10 and increased CD86 expressions, which alleviates allergic airway inflammation by suppressing Th2 polarization (127). These findings have highlighted the environment-dependent functional plasticity of TLRs in regulating pulmonary adaptive immunity.

In summary, TLRs play important roles in the regulation of pulmonary adaptive immunity. They contribute to the activation and recruitment of immune cells to establish an effective defense network against pathogens. In pathological states, aberrant TLRs activation causes excessive inflammatory response, chronic inflammation, tumor immune evasion, and autoimmune disorders. This functional plasticity of TLRs underscores the promising use of TLRs-targeted immunotherapeutic strategies for pulmonary diseases by controlling TLRs-mediated innate and adaptive immune responses.

## 5 Dysregulation of TLR networks in pulmonary pathologies

TLRs play essential roles in maintaining pulmonary homeostasis and immune and immune defense; however, their aberrant activation is implicated in various lung pathophysiological processes (**Figure 1**). Endothelial injury and interstitial fibrosis, common features in pulmonary disorders, are closely linked to dysregulated TLR signaling (128, 129). For instance, endothelial TLR2 facilitates cell injury and coagulopathy by mediating neutrophil extracellular trap (NET)-STING interactions (130). In LPS-induced ARDS, the SP1-TLR2-NF-κB axis downregulates versican V1 in lung fibroblasts, amplifying inflammation (131). TLR4 activation by LPS disrupts endothelial barrier integrity *via* Src/VE-cadherin signaling (132). Beyond bacterial ligands, TLR7 recognizes extracellular miR-146a-5p and aggravates pulmonary endothelial dysfunction in sepsis-associated ARDS (133). Additionally, TLR7/8 activation promotes endothelial injury and fibrosis, contributing to autoimmune vasculopathy (134).

TLR signaling is further influenced by gut microbiota dysbiosis. Postnatal growth restriction in extremely preterm infants predisposes to bronchopulmonary dysplasia and pulmonary hypertension, linked to microbiota-driven TLR4 activation in the lung (135). Moreover, LPS-induced TLR4 signaling desensitizes alveolar macrophages, impairing immune defense and promoting lung structural abnormalities (136).

This section examines the pathological outcomes of dysregulated TLR activation across pulmonary diseases, including infectious, allergic, inflammatory, and malignant conditions such as asthma, COPD, ILD, and lung cancer. The analysis aims to elucidate underlying molecular mechanisms and inform TLR-targeted therapeutic strategies.

### 5.1 TLRs in pulmonary infectious diseases

#### 5.1.1 TLRs in bacterial pneumonia

Infectious pneumonia poses a significant global public health challenge, with the pathogenesis intricately linked to TLR-mediated inflammatory cascades. TLRs exhibit complex molecular regulatory mechanisms that balance host defense and immunopathology (137–139). In bacterial pneumonia, the TLR family is essential for pathogen-specific recognition (**Table 2**). TLR2 recognizes peptidoglycan (PGN) and lipoproteins derived from Gram-positive bacteria, driving IL-8 secretion and neutrophil recruitment in a *S. pneumoniae* infection (140). This process is essential for pathogen clearance; however, excessive activation of TLR2 leads to acute lung injury. Notably, *Acinetobacter baumannii* activates the TLR2/NF-κB/IQGAP1 pathway *via* its outer membrane protein A, leading to the redistribution of E-cadherin in lung epithelial cells and the epithelial barrier dysfunction (141). As a result, TLR2 plays dual roles in maintaining the epithelial barrier integrity. TLR4, as the core receptor for LPS from Gram-negative bacteria, mediates inflammatory storm through activation of the MyD88/NF-κB-dependent signaling pathway (142, 143). Pathogen-induced activation of TLR4 often triggers excessive activation of NF-κB and the subsequent production of inflammatory cytokines, leading to infiltration of alveolar macrophages and neutrophils and ultimately pulmonary injury (144). In a *Staphylococcus aureus* pneumonia model, the interaction between aconitate decarboxylase 1 (ACOD1) and TLR4 exacerbates lung injury by activating NF-κB signaling (145). Natural compounds such as Anemoside B4 mitigate lung injury *via* the TLR4/MyD88 pathway (146), whereas TLR4 activation by monophosphoryl lipid A (MPLA) can synergize with antibiotics to enhance bactericidal effects (147). Additionally, other TLR-dependent therapeutic strategies are particularly noteworthy. For instance, mesenchymal stem cell-derived microvesicles (MVs) enhance the antimicrobial activity of human alveolar macrophages through TLR3 pre-activation, thereby improving the efficacy of MVs (148). TLR5 agonist flagellin exhibits broad-spectrum anti-inflammatory effects in a dual infection model of *Pseudomonas aeruginosa* and *S. pneumoniae* by inhibiting NF-κB nuclear translocation (149, 150). In a *Pseudomonas aeruginosa* infection model, the absence of TLR7 not only enhances IL-10-mediated anti-inflammatory responses, but significantly promotes bacterial clearance (151). This suggests the vital role of TLR7 in infectious pneumonia caused by Gram-negative bacteria. TLR9 specifically recognizes CpG DNA from *Prevotella* and other pathogens, which suppresses neutrophil phagocytic activity and facilitates bacterial escape from host defenses by promoting elastase release and downregulating complement C5a (152). Notably, nanoscale outer membrane vesicles secreted by Gram-negative bacteria activate lung macrophages *via* the TLR4-TRIF pathway (153).

**Table 2:** Effects of mechanisms of TLRs in immune regulation of different pulmonary diseases.

Lung diseases	TLRs	Biological effects	Molecular mechanisms	Refs.
Infectious Pneumonia	TLR2	Mediating pneumococcal clearance, neutrophil infiltration, and acute lung damage	Recognizing PGN and lipoproteins, triggering IL-8-mediated inflammation	(140)
	TLR2	Involving in A. baumannii-induced epithelial barrier dysfunction and bacterial translocation	Recognizing outer membrane protein A, activating NF-κB/IQGAP1 pathway, inducing E-cadherin redistribution	(141)
	TLR3	Mediating antiviral response and epithelial barrier damage	Promoting IFN-λ expression in lung epithelial cells	(156)
	TLR4	Mediating inflammatory lung injury in Staphylococcus aureus pneumonia	Recognizing ACOD1, activating MyD88/NF-κB pathway	(145)
	TLR4	Activating antiviral response in lung macrophages	Activating TLR4-TRIF pathway via Gram-negative OMVs	(153)
	TLR4	Mediating alveolar damage and ARDS in COVID-19	Recognizing spike protein, upregulating ACE2 and SPARCL1 expression, inducing M1 macrophage polarization	(154)
	TLR4	Facilitating Cryptococcus neoforman's immune evasion	Activating TLR4/STAT3 axis, promoting arginase-1 expression and IL-4 sensitivity in macrophages	(162)
	TLR4	Mediating persistent low-grade inflammation in Mycoplasma pneumoniae infection	Activating autophagy-NF-κB positive feedback loop inducing upregulation of TNF-α and IL-1β in macrophages	(163)
	TLR5	Anti-Pseudomonas aeruginosa and Streptococcus pneumoniae infections	Inhibition of NF-κB nuclear translocation	(149, 150)
	TLR7	TLR7 deficiency improving Pseudomonas aeruginosa clearance and mouse survival	Enhancing IL-10-mediated anti-inflammatory response	(151)
	TLR7	Inhibiting viral replication, exacerbate inflammatory lung dysfunction	Mediating IFN/ISG antiviral response	(157, 158)
	TLR9	Weakening neutrophil phagocytosis aiding E. coli to escape host defenses	Recognizing of pathogen DNA promoting elastase release and complement C5a downregulation	(152)
	TLR9	Clearing Influenza virus clearance and tissue damage		(159)
COPD	TLR2	Promoting monocyte-mediated airway inflammation	Recognizing of XPO6, activating of MyD88/NF-κB pathway, increasing expression of TNF-α, IL-6, and IL-1β	(169)
	TLR4	Mediating COPD induced by environmental particulate matter	Activating of MyD88/mTOR-autophagy, upregulating of IL-6 and CXCL1/2 in epithelium	(171)
	TLR7	Promoting mast cell degranulation	Upregulating of MMCP-6 expression	(172)
	TLR9	Inducing autoimmune persistent airway inflammation	Recognizing of NETs-DNA, activating of cGAS/TLR9/NF-κB pathway	(176)
Asthma	TLR2	Promoting Th2 cell polarization	Activating of NF-κB and JNK pathways, upregulating of TSLP expression	(170)
	TLR3	Promoting differentiation of epithelial stem cells into mucous cells	Activating of moDC/IL-33 axis, increasing IL-13 expression	(174)
	TLR3	Attenuating type 2 immune response in the lungs	Inhibiting of ILC2 differentiation via IFN-β/STAT5/GATA3 axis	(175)
	TLR4	Mediating asthma induced by environmental particulate matter	Activates MyD88/mTOR-autophagy, upregulates IL-6/CXCL1/2	(171)
	TLR5	rDCs and Tregs inhibiting asthma development	Inhibiting of TH1, TH2, and TH17 responses	(180)
	TLR7	Inducing dysregulation of innate immune responses in nasal mucosa	Upregulating of IFN-α2a, CCL3, and CCL13	(173)
IPF	TLR2/4	Promoting neutrophil infiltration and Th17 immune response	OMVs activating AMs via TLR2/4–MyD88 axis, inducing IL-17B/TNF-α network	(187)
	TLR4	Aberrant AM proliferation and autophagy–apoptosis imbalance	Activating TLR4–MyD88–NF-κB pathway	(188)
		Driving abnormal proliferation and EMT in alveolar type II epithelial cells	eNAMPT acting as DAMP ligand activating TLR4 signaling	(189)
		Creating a pro-fibrotic microenvironment, promoting macrophage M2 polarization	Physically interacting with THBS1, activating glycolytic metabolism	(190)
	TLR9	Promoting fibroblast activation, releasing inflammatory factors, establishing an "inflammation-fibrosis feedback loop"	Recognizing mtDNA, synergizing with TGF-β1; activating AHR via tryptophan metabolism	(185)
		Enhancing pulmonary epithelial pyroptosis	Binding NLRP3, amplifying caspase-1 activity	(186)
CTD-ILD	TLR3	Activating of CD4+ T cells in CADM-ILD	Recognizing of MDA5, upregulating of IL-6 expression	(197)
	TLR4	Promoting collagen synthesis and myofibroblast differentiation in SSc-ILD fibroblasts	CXCL4 enhancing TLR4 signaling by inhibiting FLI1	(195)
	TLR4	Inducing SLE-associated diffuse alveolar hemorrhage	Mediating autophagy and NETs formation	(203)
	TLR5	Inducing EMT in AEC II	Recognizing of Anti-CARP, promoting NF-κB activation	(199)
	TLR7/8	Mediating vascular remodeling abnormalities in autoimmune diseases	Regulating Th17/Treg balance	(134)
	TLR7/9	Promoting myofibroblast accumulation in SSc-ILD	Recognizing of mtDNA, activating cGAS-STING, upregulating Type I IFNs and IL-6 expression	(192)
	TLR7/9	Promoting the development of CADM-ILD	Promoting excessive production of IFN-α via TLR7/9-IRF7 pathway	(197, 198)
Hp	TLR2	Exacerbating pulmonary fibrosis	Activating TLR2–NF-κB pathway, expanding MMP14hi macrophages and releasing exosomes to enhance FMT	(204)
	TLR9	Activating lung inflammation	Activating CD11b^+^CD11c^+^ dendritic cells via the TLR9–MyD88 pathway	(205)
Silicosis	TLR4	Promoting fibroblast and alveolar epithelial cell activation	Activation of the TLR4-NF-κB/MAPK pathway, inducing pyroptosis in macrophages	(206)
		Facilitating endothelial–mesenchymal and endothelial–immune crosstalk	Galectin-3 binding TGFBR1 and TLR4, promoting FMT and NLRP3 activation	(206)
		Accelerating silicosis fibrosis progression	LPS/TLR4 signaling inducing lung microbiota dysbiosis	(207)
Lung Tumors	TLR2	Enhancing lung cancer cell migration and invasion	Activating of cAMP/AMPK/NF-κB pathway, upregulating of CCL2, IL-6, and MMP-2	(208)
	TLR2	Tumor cell senescence and myeloid cell recruitment enhancing antitumor immunity	Activating of p53-p21 pathway, leading to SASP	(209)
	TLR3	Promoting the formation of a pro-carcinogenic inflammatory microenvironment	Recognizing of L-MPs, promoting NLRP3 activation and IL-1β release	(210)
	TLR4	Enhancing tumor cell survival and metastasis	Activating of TRAF6/BECN1	(214)
	TLR4	Recruiting of mo-MDSCs and promoting of lung metastasis	Activating of CXCL10-CXCR3 and CCL12	(220)
	TLR4	Recruiting of PMN-MDSCs, promoting the establishment of a pre-metastatic niche in lung cancer	Recognizing of HSP70, activating of the Wnt5a/CXCL5/G-CSF axis	(221)
	TLR2/4	Promoting TANs recruitment during NTHi infection	Upregulating of IL-17C expression in lung epithelial cells	(216)
	TLR3/4	Promoting lung cancer invasion and metastasis	Ubiquitinating TRAF6, activating MAPK/NF-κB pathway	(215)
	TLR9	Activating CSCs	Recognizing of mtDNA, activating of Notch1/AMPK axis	(218)

#### 5.1.2 TLRs in viral pneumonia

The TLR regulatory network in viral pneumonia exhibits greater complexity (**Table 2**). The binding of the SARS-CoV-2 spike protein to TLR4 not only enhances angiotensin-converting enzyme 2 (ACE2) expression and disrupts type II alveolar cells, but induces M1 polarization *via* endothelial cell-derived secreted protein acidic and rich in cysteine-like 1 (SPARCL1) (154). Moreover, TLR4 can be modulated by extracellular vesicles (EVs)-derived miRNAs in COVID-19. In the early stage, EVs-delivering miR-146a-5p suppresses TLR4 activation to limit excessive inflammatory response. In the later stage, EVs-delivering let-7e-5p leads to more severe pulmonary inflammation and tissue damage by upregulating TLR4 expression, thereby inducing ARDS during COVID-19 infection (155). Furthermore, studies on respiratory syncytial virus and influenza virus further elucidate the dual roles of TLRs. TLR3 activation can induce an antiviral response in lung epithelial cells by promoting the expression of IFN-λ (156). However, excessive TLR3 activation can lead to epithelial barrier damage. Although TLR7-mediated IFN/ISG antiviral responses inhibit SARS-CoV-2 replication (157), they exacerbate pulmonary dysfunction in influenza A virus infection (158). Similarly, TLR9-mediated clearance of influenza virus occurs alongside tissue damage (159), suggesting that precise regulation of TLRs signal may be key to overcoming the therapeutic bottleneck in infectious pneumonia.

Current evidence has supported that the bidirectional regulation strategy of immune activation and anti-inflammation with TLR-targeted drugs exhibits unique therapeutic potential in virus-associated infectious pneumonia. The synergistic application of TLR2/6/9 agonists Pam2 CSK4 (Pam2) and CpG oligodeoxynucleotides (ODN) enhances the recruitment of pulmonary phagocytes and the cytotoxicity of natural killer cells (160). Flavonoid glycosides achieve the blockade of influenza A virus (IAV) infection by inhibiting the expression of TLR3/4/7 and the phosphorylation of NF-κB/p65 in the lung tissues of acute lung injury (ALI) mice (161). These findings indicate that TLR-targeting drugs may offer new approaches for complex viral infections.

#### 5.1.3 TLRs in fungal and mycoplasmal pneumonia

TLRs signaling also plays essential role in regulating Fungal and mycoplasma-associated infectious pneumonia (**Table 2**). *Cryptococcus neoformans* promotes the conversion of macrophages towards an IL-4-sensitive phenotype utilizing a virulence factor (CPL1) through the TLR4/STAT3 axis (162). *Mycoplasma pneumonia* is demonstrated to induce a sustained low-grade inflammatory response, characterized by upregulated TNF-α and IL-1β expression in macrophages by activating TLR4 and forming an autophagy-NF-κB positive feedback loop (163). TLR4-induced persistent inflammation drives the progression of chronic inflammatory diseases. Besides, elevated TLR2 expression in the peripheral blood of children with *Mycoplasma pneumoniae* pneumonia is positively correlated with neutrophil infiltration (164).

As evidenced above, TLRs play complicated roles in infectious pneumonia, which are involved in the initiation of host defense by the recognition of PAMPs, the inflammatory storms, and pulmonary tissue damages in infectious pneumonia due to abundant activation of TLRs signaling pathways. Targeting TLRs and the downstream signaling pathways holds great promise for the treatment of infectious pneumonia.

### 5.2 TLRs in non-infectious pulmonary diseases

#### 5.2.1 Asthma and COPD

Asthma and COPD are both classified as chronic airway inflammatory disorders, primarily characterized by inflammatory cell infiltration and the release of pro-inflammatory mediators. Clinically, patients exhibit not only significant airflow limitation but also varying degrees of airway hyperresponsiveness (165, 166). Emerging studies have demonstrated that TLRs play a pivotal role in modulating chronic airway inflammatory disorders through the crosstalk between innate and adaptive immunity (167, 168) (**Table 2**).

A previous study suggests that excessive activation of TLR2/4/7 drove airway inflammation in COPD by enhancing the nuclear export of TLR2 mediated by exportin XPO6 in monocytes, which leads to increased production of TNF-α and IL-6 through the activation of TLR2/MyD88/NF-κB pathway (169). TLR2 promotes Th2 cell polarization in asthma by thymic stromal lymphopoietin (TSLP)-mediated NF-κB and JNK signaling pathways activation in the airway epithelial cells (170). Air pollution material (PM) causes airway inflammatory disorders by inducing increased production of IL-6 and CXCL1/2 in airway epithelial cells through the TLR4/MyD88 and mTOR-autophagy signaling pathways (171). Additionally, cigarette smoke activates mast cell degranulation *via* TLR7, promoting the release of mast cell protease-6 (MMCP-6) and exacerbating emphysema in COPD (172). However, the TLR7 agonist R848 leads to the dysregulation of the innate immune response in nasal mucosa through the upregulation of IFN-α2a, CCL3, and CCL13 in asthma patients (173). Notably, TLR3 activation promotes high expression of IL-13 in type 2 innate lymphoid cells (ILC2) and alveolar macrophages, leading to airway hyperresponsiveness and increased mucus production (174). However, during the chronic phase, stimulation with the TLR3 agonist poly (I: C) inhibits ILC2 differentiation through the IFN-β/STAT5/GATA3 pathway, thereby suppressing type 2 immune response in the lung (175). In COPD, NET-derived DNA promotes NF-κB-dependent autoimmunity *via* the cGAS/TLR9 pathway, contributing to persistent airway inflammation (176). Nonetheless, TLR9 agonists have been shown to inhibit eosinophil infiltration in asthma due to the expansion of Bregs (177). Additionally, activation of TLR5 has been found to exacerbate airway inflammation in asthma (178, 179), whereas the regulatory DCs (rDCs) and Tregs can suppress TH1/TH2/TH17 responses in a TLR5-dependent manner, thereby inhibiting the development of experimental asthma (180). These findings have implicated the complicated roles of TLRs in the regulation of chronic inflammatory lung diseases, underscoring the significant challenge of achieving precise immune modulation using TLR-based therapies in the future.

#### 5.2.2 ILDs

ILDs comprise a heterogeneous group of pulmonary disorders characterized by interstitial inflammation and fibrosis, often leading to progressive dyspnea and end-stage respiratory failure. Idiopathic Pulmonary Fibrosis (IPF) is the most prevalent subtype, accounting for approximately one-third of ILD cases. Additionally, Connective Tissue Disease-associated Interstitial Lung Disease (CTD-ILD) and hypersensitivity pneumonitis (HP) are common subtypes, representing 25% and 15% of cases, respectively (181). This section focuses on elucidating the mechanisms by which TLRs drive disease initiation and progression in major ILD subtypes, including IPF, CTD-ILD, hypersensitivity pneumonitis, and silicosis.

##### 5.2.2.1 IPF

IPF is a chronic progressive ILD with unknown etiology, pathologically defined by aberrant fibroblast activation, alveolar epithelial cell dysfunction, and macrophage-driven inflammation (182). Accumulating evidence demonstrates that dysregulated TLR signaling contributes centrally to IPF pathogenesis through orchestrating inflammatory cascades, metabolic reprogramming, and fibrotic remodeling (**Table 2**).

The genetic polymorphisms of TLR3 (specifically the L412F variant) are linked to accelerated disease progression and higher mortality in IPF, underscoring the role of TLRs in phenotypic modulation (183). Fibroblast-expressed TLR9 recognizes circulating mitochondrial DNA (mtDNA) and acts synergistically with transforming growth factor-beta 1 (TGF-β1) to promote fibroblast activation, triggering the release of pro-inflammatory mediators, such as MCP-1 and IL-6 (184). This establishes a pro-fibrotic feedback loop culminating in excessive extracellular matrix (ECM) deposition (184). TLR9 also upregulates TDO2 in fibroblasts, increasing kynurenine production, which activates the AHR pathway in CD103^+^ dendritic cells and enhances IL-6-driven inflammation and fibrosis (185). Additionally, epithelial TLR9 engages the NLRP3 inflammasome to promote caspase-1-mediated pyroptosis, further contributing to IPF pathogenesis (186). These findings collectively underscore the critical role of TLR9 in the regulation of pulmonary fibrosis.

Host-microbe interactions also promote fibrotic in IPF *via* TLR2/4. Dysbiosis-associated outer membrane vesicles (OMVs), particularly derived from *Bacteroides* and *Prevotella* species, activate AMs *via* the TLR2/4-MyD88 signaling axis, thereby inducing a profibrotic network involving IL-17B and TNF-α (187). This upregulates neutrophil chemokines (e.g., G-CSF, CXCL1, CXCL2) and Th17 differentiation genes (e.g., IL-6, Saa1/2), fostering neutrophil infiltration and Th17 responses that accelerate fibrosis (187). Unlike classical autoimmune ILDs, IPF appears driven primarily by DAMPs and microbiota-derived ligands rather than autoantibody-mediated TLR activation.

In addition, the TLR4 signaling pathway plays multiple roles in IPF. Activation of the TLR4-MyD88-NF-κB axis in AMs leads to aberrant AM proliferation and disruption of the "autophagy-apoptosis" equilibrium, significantly exacerbating disease progression (188). Elevated eNAMPT-a DAMP and TLR4 ligand—in IPF patients correlates with severity and drives alveolar type II cell proliferation and EMT *via* TLR4, facilitating pathological remodeling (189). TLR4 also interacts with THBS1 to induce M2 macrophage polarization and glycolytic activation, establishing a pro-fibrotic microenvironment (190).

In summary, TLRs integrate signals from microorganisms, DAMPs, and cellular stress through key pathways, including MyD88, NF-κB, NLRP3, and metabolic reprogramming, forming a central bridge between innate immunity, chronic inflammation, and fibrosis in IPF. Targeted inhibition of specific TLRs or downstream effectors may offer promising therapeutic strategies for IPF.

##### 5.2.2.2 CTD-ILD

CTD-ILD is a significant complication of systemic autoimmune disorders, such as rheumatoid arthritis, systemic sclerosis, and dermatomyositis. The pathogenesis of CTD-ILD is closely linked to dysfunction of alveolar type II epithelial cells (AEC II), inflammatory cascade activation, and aberrant fibroblast activation (191). TLRs play a crucial role in immune activation and fibrosis progression of CTD-ILD by recognizing DAMPs or PAMPs (**Table 2**).

TLR family is dysregulated in lung tissues of patients with systemic sclerosis-associated interstitial lung disease (SSc-ILD) (129). Extracellular vesicle-delivered mtDNA can activate the cGAS/STING pathway *via* TLR9, promoting the secretion of type I IFNs and IL-6, thereby driving the accumulation of α-smooth muscle actin (α-SMA)^+^ myofibroblasts (192). TLR8 is significantly upregulated in monocytes during the early stage of SSc-ILD (193). However, declined expression of TLR8 is well demonstrated in the late stages of the of SSc-ILD (129). In addition, TLR/CXCL4 signaling exacerbates endothelial cell activation and fibrosis by inhibiting the transcription factor FLI1 (194). As a small-molecule inhibitor of TLR4, TAK242 can suppress collagen synthesis in fibroblasts, offering a potential therapeutic strategy for SSc-ILD (195). The anti-melanoma differentiation-associated gene 5 (MDA5) antibody is positively associated with amyopathic dermatomyositis-associated interstitial lung disease (CADM-ILD) (196, 197). Overactivation of the TLR7/9-IRF7 axis leads to aberrant production of IFN-α, while MDA5 autoantibodies promote IL-6 secretion by activating CD4^+^ T cells *via* TLR3 (197, 198). The therapeutic efficacy of anti-CD4 antibodies and IL-6 receptor antagonists further validates the critical role of TLR7/9-IRF7 axis (197). Additionally, the interaction between carbamylated TLR5 on AEC II cells and anti-carbamylated protein (anti-CarP) antibodies can induce nuclear translocation of NF-κB and promote EMT in AEC II, thereby accelerating fibrosis (199). Systemic lupus erythematosus (SLE) is characterized by significant mitochondrial dysfunction, where mitochondrial damage releases mtRNA that can be recognized by TLR7, subsequently triggering type I IFN responses (200–202). Notably, TLR7/8 can participate in the vascular remodeling abnormalities seen in autoimmune diseases by regulating the Th17/Treg balance, a process that is associated with complications such as pulmonary arterial hypertension (134). Additionally, TLR4-mediated autophagy and NET formation have been linked to diffuse alveolar hemorrhage in SLE (203). Therefore, TLRs play a crucial role in the regulation of CTD-ILD, serving as potential targets for disease treatment.

In summary, TLRs play a crucial role in the development and progression of CTD-ILD by regulating fibroblast activation, inflammatory cytokine release, and autoantibody production. These findings not only highlight the central role of TLRs in CTD-ILD, but provide new insights into the mechanisms of autoimmune diseases and the explanation of targeted therapies.

##### 5.2.2.3 HP and Other ILDs

The pathogenesis and progression of HP and other ILDs are closely associated with immune and inflammatory responses mediated by TLR signaling. Although the mechanisms vary considerably across different etiologies and experimental models, certain common pathways have emerged (**Table 2**).

In a model of HP induced by *Saccharopolyspora rectivirgula* antigen, activation of the TLR2-NF-κB signaling pathway promotes the expansion of matrix metalloproteinase-14 (MMP14) high expressed macrophage subset and the release of exosomes (204). This subset enhances fibroblast-to-myofibroblast transition (FMT), thereby exacerbating pulmonary fibrosis (204). In contrast, in mycobacterium-induced HP, the activation of CD11b^+^CD11c^+^ dendritic cells *via* the TLR9-MyD88 pathway serves as a key mechanism in the development of lung inflammation. This process occurs independent of pathogen infectivity, highlighting the specific role of TLR9 in non-infectious immune responses (205).

In a silica (SiO_2_)-induced model of silicosis, SiO_2_ particles activate the TLR4-NF-κB/MAPK signaling pathway in macrophages, leading to macrophage pyroptosis and fibroblasts and alveolar epithelial cells activation, significantly amplifying pulmonary inflammation and fibrosis (206). Galectin-3 (Gal3) derived from senescent endothelial cells simultaneously engages TGFBR1 on fibroblasts and TLR4 on macrophages, thereby mediating endothelial-mesenchymal and endothelial-immune crosstalk (206). This interaction synergistically promotes both FMT and NLRP3 inflammasome activation, contributing to the progression of interstitial lung pathology (206). Moreover, dysbiosis of the lung microbiota resulting from LPS/TLR4 activation has also been found to promote the progression of silica-induced fibrosis (207), underscoring the key role of microbe-host interactions in environmentally-related lung diseases.

In summary, TLR-mediated activation of downstream cascades, including NF-κB, MAPK, and MyD88, orchestrates multicellular crosstalk among macrophages, dendritic cells, fibroblasts, and endothelial cells in both hypersensitivity pneumonitis and silicosis, thereby driving coordinated inflammatory and fibrotic responses. These insights not only underscore the centrality of TLR signaling networks in the pathogenesis of interstitial lung diseases but also highlight the therapeutic potential of targeting specific TLRs or their effector pathways to attenuate fibrosis progression.

#### 5.2.3 Lung carcinomas

TLRs exert complex effects on the initiation, progression, and immune microenvironment of lung cancer. TLR2 enhances lung cancer cell migration and invasion by promoting the expression of CCL2, IL-6, and MMP-2 through the cAMP-AMPK-TAK1 signaling axis (208). Nonetheless, TLR2 also exhibits anti-cancer effects under specific conditions. In NSCLC, TLR2 activation induces tumor cell senescence by activating the p53-p21 pathway and promoting the expression of pro-inflammatory senescence-associated secretory phenotype (SASP) (209). Besides, TLR3 contributes to the establishment of a pro-tumorigenic inflammatory microenvironment to promote lung cancer progression *via* NLRP3 inflammasome activation and subsequent IL-1β release (210). Autophagy promotes tumor cell survival and migration (211). Accumulated studies have suggested TLRs are involved in the regulation of autophagy and the pre-metastatic niche (212, 213). TLR4 can enhance tumor survival and metastasis by inducing autophagy *via* the TRAF6-BECN1 axis (214). TLR3/4 activation results in the upregulation of chemokines CCL2/MCP-1 and immunosuppressive factors VEGFA and MMP2, which collectively promotes lung cancer invasion and metastasis through the adaptor protein TICAM1/TRIF and the activation of downstream MAPK/NF-κB signaling pathway (215). Nontypeable *Haemophilus influenzae* (NTHi) induces lung epithelial cells to secrete IL-17C *via* TLR2/4 signaling, thereby promoting lung cancer progression (216). Moreover, microbial metabolites, such as FFAR, can inhibit lung cancer progression through functional competition with TLR2/4 (208). Additionally, the activation of endogenous TLR7 within tumors can recruit MDSCs, which facilitates EMT and the metastasis of lung adenocarcinoma (217). It has been demonstrated that mitophagy-released mtDNAs activate cancer stem-like cells (CSCs) *via* the TLR9-Notch1-AMPK axis, leading to chemoresistance and tumor recurrence (218). These findings indicate that TLR signaling play critical roles in tumorigenesis, metastasis, cancer resistance to therapy and microbial interaction in lung carcinomas (**Table 2**).

The pre-metastatic niche is a microenvironment created by the primary tumor in secondary organs and tissues that facilitates subsequent metastasis (219). TLRs are also involved in lung cancer metastasis by regulating the pre-metastatic niche. In metastatic lung cancer, TLR4 in alveolar macrophages promotes pulmonary metastasis by recruiting monocyte-derived myeloid-derived suppressor cells (mo-MDSCs) and activating the CXCL10-CXCR3/CCL12 axis (220). Heat shock protein 70 (HSP70) recruits polymorphonuclear myeloid-derived suppressor cells (PMN-MDSCs) through the TLR4-Wnt5a-CXCL5/G-CSF axis, contributing to the establishment of a pre-metastatic niche and resistance to immunotherapy in lung cancer (221). Additionally, the tumor-derived exosomal RNAs promote lung pre-metastatic niche formation *via* activating TLR3, driving neutrophil infiltration and the establishment of a pre-metastatic microenvironment (222). Accordingly, targeting TLRs and TLR signaling may represent a novel immunotherapeutic strategy in lung cancer.

## 6 Overview of clinical studies on TLRs- and TLRs signaling-based drugs

In recent years, significant advance has been made in therapeutic drugs targeting TLRs and the TLRs signaling pathways, which holds promising therapeutic potentials in lung cancer, asthma, and COPD (**Table 3**) (223).

**Table 3:** Overview of clinical studies on TLRs and TLR signaling pathways.

Category	Drug	Target	Disease	Mechanism	Refs.
TLR Agonists	CADI-05	TLR2	Lung Tumors	Activation of anti-angiogenic phenotype in TANs improves patient survival	(230)
	BCG-CWS	TLR2/4		TLR2/4-Mφ/APC axis induces IL-12/IL-18 secretion and enhances IFN-γ production	(229)
	Resiquimod(R848)	TLR7/8		Establishment of antiviral immunity in respiratory mucosa	(233)
	IMO-2055	TLR9		Combination of Erlotinib and Bevacizumab enhances antitumor immune response in patients	(203)
	DV281			Combination with Nivolumab enhances antitumor immune response in patients	(131)
	CpG ODN(K3)			Increased IFN-α secretion and expansion of T-bet+ CD8+ T cells prolong patient FPS	(226)
	PF-3512676			Combination chemotherapy (Paclitaxel/Cisplatin or Gemcitabine/Cisplatin) leads to immune exhaustion	(227, 228)
TLR Inhibitors	CNTO3157	TLR3	COPD	Reduction of airway hyperresponsiveness	(234)
	TNFAIP3 (A20) mimetic peptide	TLR4	Asthma	Inhibition of TLR4/TRAF6/NF-κB pathway	(235)

### 6.1 TLR agonists

In clinical trials, the TLR9 agonist IMO-2055 in combination with erlotinib and bevacizumab (no. NCT00633529), as well as DV281 combined with nivolumab (no. NCT03326752), demonstrates favorable tolerability and enhanced antitumor immune response in patients with advanced NSCLC (224, 225). As TLR9 agonist, CpG ODN (K3) prolongs the survival of lung cancer patients by inducing IFN-α secretion and the expansion of T-bet^+^ CD8^+^ T cells (no. UMIN-000023276) (226). However, targeted TLR activation aimed at enhancing antitumor or anti-pathogen immunity inherently may inadvertently aggravate pre-existing inflammatory conditions. For example, the TLR9 agonist PF-3512676 in combination with chemotherapy regimens (paclitaxel/carboplatin or gemcitabine/cisplatin) showed limited efficacy in improving overall survival in lung cancer patients, accompanied by risks of immune exhaustion, highlighting the hazards of excessive or non-specific TLR activation (no. NCT00254891) (227, 228).

TLR2/4 agonists exhibit unique value in reshaping the immune microenvironment. Bacillus Calmette-Guérin-cell wall skeleton (BCG-CWS) leads to tumor regression in lung cancer patients by inducing the secretion of IL-12 and IL-18 *via* the TLR2/4-macrophage (Mφ)/antigen-presenting cell (APC) axis (229). Besides, the TLR-2 agonist CADI-05 activates an anti-angiogenic phenotype in TANs from patients with squamous cell lung carcinoma (no. NTC00680940) (230). However, the efficacy of TLR agonists is significantly influenced by diverse factors. In smokers and COPD patients, the expression of TLR2 in alveolar macrophage is significantly reduced (231). Nicotine restores TLR2/9 responsiveness by upregulating CD4^+^CD25^+^FoxP3^+^ Tregs (no. NCT00701207) (232). This variability underscores the risk of failure inherent in sole reliance on TLR-targeted agonist therapies and highlights their potential unsafety in non-responsive patient subpopulations. The central challenge for future research lies in precisely identifying patient cohorts who benefit from TLR modulation, defining the therapeutic window, and advancing biomarker-driven personalized therapy to balance efficacy and safety. Furthermore, the antiviral immune model established by the TLR7/8 agonist R848 in the respiratory mucosa offers new perspectives for combined interventions targeting virus-associated lung tumors (no. NCT02090374) (233).

### 6.2 TLR inhibitors and TLRs signaling-targeted drugs

Significant progress has been made in the development of TLRs inhibitors. The TLR3 monoclonal antibody CNTO3157 reduces rhinovirus-induced airway hyperresponsiveness in healthy subjects; however, it shows limited efficacy in improving symptoms in patients with COPD (no. NCT01704040) (234). In asthma, TNFAIP3 (A20) mimetic peptides reduce the frequency of acute exacerbations in asthmatic children from urban areas by inhibiting the TLR4/TRAF6/NF-κB pathway (235). These findings suggest that targeting TLRs and TLR signaling could be an effective method to manage asthma symptoms and improve the quality of life in affected populations. Thus, TLR-targeted therapies are promising in the treatment of pulmonary diseases. A thorough understanding of the regulatory immune networks governing TLRs and TLR signaling may provide novel insight into the exploration of precision medicine strategies in pulmonary diseases. Chronic pulmonary diseases, such as COPD and asthma, involve persistent inflammation and immune dysregulation. In such settings, TLR agonists risk amplifying pathological inflammation, potentially leading to adverse events and clinical worsening. Conversely, TLR antagonists may systemically inhibit essential TLR pathways, compromising anti-infective immunity and increasing susceptibility to opportunistic infections—particularly in immunocompromised individuals, including those with cancer or chronic respiratory conditions.

## 7 Conclusions and future directions

TLRs are essential for the maintenance of lung homeostasis by regulating epithelial barrier integrity, endothelial cell activity, microbial communities balance and immune cells functions. The well-established immune network by TLRs and TLR signaling pathways plays a pivotal role in pathogen clearance and the initiation of adaptive immunity. However, the recognition of PAMPs/DAMPs by TLRs function as a double-edged sword. Excessive activation of TLRs signaling can disrupt the immune balance, leading to pathogen escape, abundant inflammation, tissue damage, and malignant transformation. Currently, there are increasing clinical studies investigating the efficacy of TLRs- and TLRs signaling-based therapies in pulmonary diseases, including agonists and inhibitors. Most importantly, the functions and roles of TLRs in lung immunity remain not fully understood. It is of great importance to elucidate the involvement of TLRs and TLR signaling network in the onset and progression of lung diseases, including infections, fibrosis, malignancies, and immune disorders. More future clinical studies are warranted to explore the optimized therapeutic strategies targeting TLRs and TLR signaling in pulmonary diseases.

## Glossary

PRRs: pattern recognition receptors
PAMPs: pathogen-associated molecular patterns
DAMPs: danger-associated molecular patterns
IRFs: interferon regulatory factors
TM: transmembrane
TIRAP TIR: domain-containing adaptor protein
LRRs: leucine-rich repeats
MD2: myeloid differentiation factor 2
dsRNA: double-stranded RNA
MyD88: myeloid differentiation factor 88
IRAK4: interleukin-1 receptor-associated kinase 4
RIP1: receptor-interacting protein 1
TRADD: TNFR-associated death domain protein
ERK1/2: extracellular signal-regulated kinases 1 and 2
CINC: cytokine-induced neutrophil chemoattractant
MALP-2: macrophage-activating lipopeptide 2 kDa
GM-CSF: granulocyte-macrophage colony-stimulating factor
PAH: pulmonary arterial hypertension
BMPR2: bone morphogenetic protein receptor II
PAH: pulmonary arterial hypertension
NETs: neutrophil extracellular traps
ARDS: acute respiratory distress syndrome
PFS: progression-free survival
RP105: Radioprotective 105 kDa protein
PI3K: phosphoinositide 3-kinas
Mtb: Mycobacterium tuberculosis
MPLA: monophosphoryl lipid A
ROS: reactive oxygen species
NK: natural killer
TME: tumor microenvironment
TANs: Tumor-associated neutrophils
NSCLC: non-small cell lung cancer
MDSCs: myeloid-derived suppressor cells
Tregs: regulatory T cells
SSc: systemic sclerosis
Bregs: regulatory B cells
CADM: Clinically Amyopathic Dermatomyositis
SLE: Systemic Lupus, Erythematosus
CTD-ILD: Connective Tissue Disease-Associated Interstitial Lung Disease
SPARCL1: Secreted Protein Acidic and Rich in Cysteine-Like 1
ACOD1: Aconitate Decarboxylase 1
CPL1: Cell Wall Protein 1
STAT3: Signal Transducer and Activator of Transcription 3
mtDNA/RNA: Mitochondrial DNA/RNA
CpG DNA: Cytosine-phosphate-Guanine DNA
NE: Neutrophil Elastase
XPO6: Exportin 6
mTOR: Mechanistic Target of Rapamycin
CCL3/13: C-C Motif Chemokine Ligand 3/13
CXCL1/2: C-X-C Motif Chemokine Ligand 1/2
CXCR3 C-X-C: Motif Chemokine Receptor 3
cGAS: Cyclic GMP-AMP Synthase
JNK: c-Jun N-terminal Kinase
TSLP: Thymic Stromal Lymphopoietin
moDC: Monocyte-Derived Dendritic Cell
GATA3: GATA Binding Protein 3
rDCs: Regulatory Dendritic Cells
TH1/TH2/TH17: T Helper 1/2/17 Cel
Breg: Regulatory B Cell
α-SMA: Alpha-Smooth Muscle Actin
MDA5: Melanoma Differentiation-Associated Protein 5
Anti Carp: Anti-Citrullinated Protein Antibody
EMT: Epithelial-Mesenchymal Transition
Notch1: Neurogenic Locus Notch Homolog Protein 1
CSC: Cancer Stem Cell
G-CSF: Granulocyte Colony-Stimulating Factor
PMN: Polymorphonuclear Leukocyte
SASP: Senescence-Associated Secretory Phenotype
L-MPs: Large Membrane Particles
TRAF6: TNF Receptor-Associated Factor 6
MCP-1: Monocyte Chemoattractant Protein-1
VEGFA: Vascular Endothelial Growth Factor A
BECN1: Beclin 1
HSP 70: Heat Shock Protein 70

## References

[1] Nusslein-Volhard C . The toll gene in drosophila pattern formation. Trends Genet. (2022) 38:231–45. doi: 10.1016/j.tig.2021.09.006, PMID: 34649739

[2] Gay NJ Keith FJ . Drosophila toll and il-1 receptor. Nature. (1991) 351:355–6. doi: 10.1038/351355b0, PMID: 1851964

[3] Medzhitov R Preston-Hurlburt P Janeway CA Jr . A human homologue of the drosophila toll protein signals activation of adaptive immunity. Nature. (1997) 388:394–7. doi: 10.1038/41131, PMID: 9237759

[4] Kawai T Ikegawa M Ori D Akira S . Decoding toll-like receptors: recent insights and perspectives in innate immunity. Immunity. (2024) 57:649–73. doi: 10.1016/j.immuni.2024.03.004, PMID: 38599164

[5] Akira S Uematsu S Takeuchi O . Pathogen recognition and innate immunity. Cell. (2006) 124:783–801. doi: 10.1016/j.cell.2006.02.015, PMID: 16497588

[6] Asami J Shimizu T . Structural and functional understanding of the toll-like receptors. Protein Sci. (2021) 30:761–72. doi: 10.1002/pro.4043, PMID: 33576548 PMC7980524

[7] Bzówka M Bagrowska W Góra A . Recent advances in studying toll-like receptors with the use of computational methods. J Chem Inf Model. (2023) 63:3669–87. doi: 10.1021/acs.jcim.3c00419, PMID: 37285179 PMC10302489

[8] Kagan JC Su T Horng T Chow A Akira S Medzhitov R . Tram couples endocytosis of toll-like receptor 4 to the induction of interferon-beta. Nat Immunol. (2008) 9:361–8. doi: 10.1038/ni1569, PMID: 18297073 PMC4112825

[9] Ippagunta SK Pollock JA Sharma N Lin W Chen T Tawaratsumida K . Identification of toll-like receptor signaling inhibitors based on selective activation of hierarchically acting signaling proteins. Sci Signal. (2018) 11:eaaq1077. doi: 10.1126/scisignal.aaq1077, PMID: 30108181 PMC7195875

[10] Gao F Pang J Lu M Liu Z Wang M Ke X . Nile tilapia tlr3 recruits myd88 and trif as adaptors and is involved in the nf-kappab pathway in the immune response. Int J Biol Macromol. (2022) 218:878–90. doi: 10.1016/j.ijbiomac.2022.07.201, PMID: 35908672

[11] Ermolaeva MA Michallet MC Papadopoulou N Utermöhlen O Kranidioti K Kollias G . Function of tradd in tumor necrosis factor receptor 1 signaling and in trif-dependent inflammatory responses. Nat Immunol. (2008) 9:1037–46. doi: 10.1038/ni.1638, PMID: 18641654

[12] Fitzgerald KA Rowe DC Barnes BJ Caffrey DR Visintin A Latz E . Lps-tlr4 signaling to irf-3/7 and nf-kappab involves the toll adapters tram and trif. J Exp Med. (2003) 198:1043–55. doi: 10.1084/jem.20031023, PMID: 14517278 PMC2194210

[13] Kawai T Sato S Ishii KJ Coban C Hemmi H Yamamoto M . Interferon-alpha induction through toll-like receptors involves a direct interaction of irf7 with myd88 and traf6. Nat Immunol. (2004) 5:1061–8. doi: 10.1038/ni1118, PMID: 15361868

[14] Zhang E Ma Z Lu M . Contribution of T- and B-cell intrinsic toll-like receptors to the adaptive immune response in viral infectious diseases. Cell Mol Life Sci. (2022) 79:547. doi: 10.1007/s00018-022-04582-x, PMID: 36224474 PMC9555683

[15] Yamamoto M Sato S Hemmi H Hoshino K Kaisho T Sanjo H . Role of adaptor trif in the myd88-independent toll-like receptor signaling pathway. Science. (2003) 301:640–3. doi: 10.1126/science.1087262, PMID: 12855817

[16] Le J Kulatheepan Y Jeyaseelan S . Role of toll-like receptors and nod-like receptors in acute lung infection. Front Immunol. (2023) 14:1249098. doi: 10.3389/fimmu.2023.1249098, PMID: 37662905 PMC10469605

[17] Lim JS Jeon EJ Go HS Kim HJ Kim KY Nguyen TQT . Mucosal tlr5 activation controls healthspan and longevity. Nat Commun. (2024) 15:46. doi: 10.1038/s41467-023-44263-2, PMID: 38167804 PMC10761998

[18] Asano T Boisson B Onodi F Matuozzo D Moncada-Velez M Maglorius Renkilaraj MRL . X-linked recessive tlr7 deficiency in ~1% of men under 60 years old with life-threatening covid-19. Sci Immunol. (2021) 6:eabl4348. doi: 10.1126/sciimmunol.abl4348, PMID: 34413140 PMC8532080

[19] Schaunaman N Nichols T Cervantes D Hartsoe P Ferrington DA Chu HW . The effect of a tlr3 agonist on airway allergic inflammation and viral infection in immunoproteasome-deficient mice. Viruses. (2024) 16:1384. doi: 10.3390/v16091384, PMID: 39339860 PMC11437510

[20] Mubarak RA Roberts N Mason RJ Alper S Chu HW . Comparison of pro- and anti-inflammatory responses in paired human primary airway epithelial cells and alveolar macrophages. Respir Res. (2018) 19:126. doi: 10.1186/s12931-018-0825-9, PMID: 29940963 PMC6020222

[21] Pedicillo MC De Stefano IS Zamparese R Barile R Meccariello M Agostinone A . The role of toll-like receptor-4 in macrophage imbalance in lethal covid-19 lung disease, and its correlation with galectin-3. Int J Mol Sci. (2023) 24:13259. doi: 10.3390/ijms241713259, PMID: 37686069 PMC10487501

[22] Cao J Xu R Geng Y Xu S Guo M . Exposure to polystyrene microplastics triggers lung injury *via* targeting toll-like receptor 2 and activation of the nf-kappab signal in mice. Environ pollut. (2023) 320:121068. doi: 10.1016/j.envpol.2023.121068, PMID: 36641069

[23] Medzhitov R Janeway CA Jr. Decoding the patterns of self and nonself by the innate immune system. Science. (2002) 296:298–300. doi: 10.1126/science.1068883, PMID: 11951031

[24] Sha Q Truong-Tran AQ Plitt JR Beck LA Schleimer RP . Activation of airway epithelial cells by toll-like receptor agonists. Am J Respir Cell Mol Biol. (2004) 31:358–64. doi: 10.1165/rcmb.2003-0388OC, PMID: 15191912

[25] Petrikin JE Gaedigk R Leeder JS Truog WE . Selective toll–like receptor expression in human fetal lung. Pediatr Res. (2010) 68:335–8. doi: 10.1203/PDR.0b013e3181ed1134, PMID: 20581745 PMC2967298

[26] Armstrong L Medford AR Uppington KM Robertson J Witherden IR Tetley TD . Expression of functional toll-like receptor-2 and -4 on alveolar epithelial cells. Am J Respir Cell Mol Biol. (2004) 31:241–5. doi: 10.1165/rcmb.2004-0078OC, PMID: 15044215

[27] Guillot L Medjane S Le-Barillec K Balloy V Danel C Chignard M . Response of human pulmonary epithelial cells to lipopolysaccharide involves toll-like receptor 4 (Tlr4)-dependent signaling pathways: evidence for an intracellular compartmentalization of tlr4. J Biol Chem. (2004) 279:2712–8. doi: 10.1074/jbc.M305790200, PMID: 14600154

[28] Jiang D Liang J Fan J Yu S Chen S Luo Y . Regulation of lung injury and repair by toll-like receptors and hyaluronan. Nat Med. (2005) 11:1173–9. doi: 10.1038/nm1315, PMID: 16244651

[29] O'Neill LA . Tlrs play good cop, bad cop in the lung. Nat Med. (2005) 11:1161–2. doi: 10.1038/nm1105-1161, PMID: 16270071

[30] Jiang D Liang J Noble PW . Hyaluronan in tissue injury and repair. Annu Rev Cell Dev Biol. (2007) 23:435–61. doi: 10.1146/annurev.cellbio.23.090506.123337, PMID: 17506690

[31] Liang J Zhang Y Xie T Liu N Chen H Geng Y . Hyaluronan and tlr4 promote surfactant-protein-C-positive alveolar progenitor cell renewal and prevent severe pulmonary fibrosis in mice. Nat Med. (2016) 22:1285–93. doi: 10.1038/nm.4192, PMID: 27694932 PMC5503150

[32] Augustin HG Koh GY . A systems view of the vascular endothelium in health and disease. Cell. (2024) 187:4833–58. doi: 10.1016/j.cell.2024.07.012, PMID: 39241746

[33] Phelan P Merry HE Hwang B Mulligan MS . Differential toll-like receptor activation in lung ischemia reperfusion injury. J Thorac Cardiovasc Surg. (2015) 149:1653–61. doi: 10.1016/j.jtcvs.2015.02.045, PMID: 25911179 PMC4464981

[34] Grote K Schuett H Salguero G Grothusen C Jagielska J Drexler H . Toll-like receptor 2/6 stimulation promotes angiogenesis *via* gm-csf as a potential strategy for immune defense and tissue regeneration. Blood. (2010) 115:2543–52. doi: 10.1182/blood-2009-05-224402, PMID: 20056792

[35] Farkas D Thompson AAR Bhagwani AR Hultman S Ji H Kotha N . Toll-like receptor 3 is a therapeutic target for pulmonary hypertension. Am J Respir Crit Care Med. (2019) 199:199–210. doi: 10.1164/rccm.201707-1370OC, PMID: 30211629 PMC6353001

[36] Bhagwani AR Ali M Piper B Liu M Hudson J Kelly N . A P53-tlr3 axis ameliorates pulmonary hypertension by inducing bmpr2 *via* irf3. iScience. (2023) 26:105935. doi: 10.1016/j.isci.2023.105935, PMID: 36685041 PMC9852960

[37] Kim SJ Shan P Hwangbo C Zhang Y Min JN Zhang X . Endothelial toll-like receptor 4 maintains lung integrity *via* epigenetic suppression of P16(Ink4a). Aging Cell. (2019) 18:e12914. doi: 10.1111/acel.12914, PMID: 30790400 PMC6516428

[38] Marquant Q Laubreton D Drajac C Mathieu E Bouguyon E Noordine ML . The microbiota plays a critical role in the reactivity of lung immune components to innate ligands. FASEB J. (2021) 35:e21348. doi: 10.1096/fj.202002338R, PMID: 33715218

[39] Lipinski JH Falkowski NR Huffnagle GB Erb-Downward JR Dickson RP Moore BB . Toll-like receptors, environmental caging, and lung dysbiosis. Am J Physiol Lung Cell Mol Physiol. (2021) 321:L404–L15. doi: 10.1152/ajplung.00002.2021, PMID: 34159791 PMC8410110

[40] Pantaleon Garcia J Hinkle KJ Falkowski NR Evans SE Dickson RP . Selective modulation of the pulmonary innate immune response does not change lung microbiota in healthy mice. Am J Respir Crit Care Med. (2021) 204:734–6. doi: 10.1164/rccm.202104-0836LE, PMID: 34153197 PMC8521706

[41] Huang Y Ma SF Espindola MS Vij R Oldham JM Huffnagle GB . Microbes are associated with host innate immune response in idiopathic pulmonary fibrosis. Am J Respir Crit Care Med. (2017) 196:208–19. doi: 10.1164/rccm.201607-1525OC, PMID: 28157391 PMC5519968

[42] Segal LN Clemente JC Tsay JC Koralov SB Keller BC Wu BG . Enrichment of the lung microbiome with oral taxa is associated with lung inflammation of a th17 phenotype. Nat Microbiol. (2016) 1:16031. doi: 10.1038/nmicrobiol.2016.31, PMID: 27572644 PMC5010013

[43] Kohl L Hayek I Daniel C Schulze-Lührmann J Bodendorfer B Lührmann A . Myd88 is required for efficient control of coxiella burnetii infection and dissemination. Front Immunol. (2019) 10:165. doi: 10.3389/fimmu.2019.00165, PMID: 30800124 PMC6376249

[44] Sheahan T Morrison TE Funkhouser W Uematsu S Akira S Baric RS . Myd88 is required for protection from lethal infection with a mouse-adapted sars-cov. PloS Pathog. (2008) 4:e1000240. doi: 10.1371/journal.ppat.1000240, PMID: 19079579 PMC2587915

[45] Dudek M Puttur F Arnold-Schrauf C Kühl AA Holzmann B Henriques-Normark B . Lung epithelium and myeloid cells cooperate to clear acute pneumococcal infection. Mucosal Immunol. (2016) 9:1288–302. doi: 10.1038/mi.2015.128, PMID: 26627460 PMC4990776

[46] Patel S Tucker HR Gogoi H Mansouri S Jin L . Cgas-sting and myd88 pathways synergize in ly6c(Hi) monocyte to promote streptococcus pneumoniae-induced late-stage lung ifnγ Production. Front Immunol. (2021) 12:699702. doi: 10.3389/fimmu.2021.699702, PMID: 34512626 PMC8427188

[47] Nandi BR Patra B Radhakrishnan GK . A chimeric peptide derived from a bacterial effector protein attenuates tlr-2/4-mediated production of pro-inflammatory cytokines and enhances the cellular availability of gentamicin. J Inflammation Res. (2025) 18:10751–75. doi: 10.2147/jir.S526902, PMID: 40823350 PMC12350569

[48] Jeyaseelan S Young SK Yamamoto M Arndt PG Akira S Kolls JK . Toll/Il-1r Domain-Containing Adaptor Protein (Tirap) Is a Critical Mediator of Antibacterial Defense in the Lung against Klebsiella Pneumoniae but Not Pseudomonas Aeruginosa. J Immunol. (2006) 177:538–47. doi: 10.4049/jimmunol.177.1.538, PMID: 16785551

[49] Jeyaseelan S Manzer R Young SK Yamamoto M Akira S Mason RJ . Toll-il-1 receptor domain-containing adaptor protein is critical for early lung immune responses against escherichia coli lipopolysaccharide and viable escherichia coli. J Immunol. (2005) 175:7484–95. doi: 10.4049/jimmunol.175.11.7484, PMID: 16301656

[50] Lai D Zhu K Li S Xiao Y Xu Q Sun Y . Sars-cov-2 N protein triggers acute lung injury *via* modulating macrophage activation and infiltration in *in vitro* and *in vivo*. J Inflammation Res. (2023) 16:1867–77. doi: 10.2147/jir.S405722, PMID: 37143821 PMC10153437

[51] Chen P Zou Y Wang X Chen Z Dong K Yang J . Discovery of novel myd88 inhibitor A5s to alleviate acute lung injury with favorable drug-like properties. J Med Chem. (2024) 67:22263–81. doi: 10.1021/acs.jmedchem.4c02401, PMID: 39644263

[52] Zhu W Luo W Han J Zhang Q Ji L Samorodov AV . Schisandrin B protects against lps-induced inflammatory lung injury by targeting myd88. Phytomedicine. (2023) 108:154489. doi: 10.1016/j.phymed.2022.154489, PMID: 36270224

[53] Shi MM Zhu YG Yan JY Rouby JJ Summah H Monsel A . Role of mir-466 in mesenchymal stromal cell derived extracellular vesicles treating inoculation pneumonia caused by multidrug-resistant pseudomonas aeruginosa. Clin Transl Med. (2021) 11:e287. doi: 10.1002/ctm2.287, PMID: 33463070 PMC7805403

[54] Kiripolsky J Kasperek EM Zhu C Li QZ Wang J Yu G . Tissue-specific activation of myd88-dependent pathways governs disease severity in primary sjögren's syndrome. J Autoimmun. (2021) 118:102608. doi: 10.1016/j.jaut.2021.102608, PMID: 33596533 PMC8299268

[55] Totura AL Whitmore A Agnihothram S Schäfer A Katze MG Heise MT . Toll-like receptor 3 signaling *via* trif contributes to a protective innate immune response to severe acute respiratory syndrome coronavirus infection. mBio. (2015) 6:e00638-15. doi: 10.1128/mBio.00638-15, PMID: 26015500 PMC4447251

[56] Pivniouk V Gimenes-Junior JA Ezeh P Michael A Pivniouk O Hahn S . Airway administration of om-85, a bacterial lysate, blocks experimental asthma by targeting dendritic cells and the epithelium/il-33/ilc2 axis. J Allergy Clin Immunol. (2022) 149:943–56. doi: 10.1016/j.jaci.2021.09.013, PMID: 34560105 PMC8901455

[57] Cristaldi M Buscetta M Cimino M La Mensa A Giuffrè MR Fiore L . Caspase-8 activation by cigarette smoke induces pro-inflammatory cell death of human macrophages exposed to lipopolysaccharide. Cell Death Dis. (2023) 14:773. doi: 10.1038/s41419-023-06318-6, PMID: 38007509 PMC10676397

[58] Chen K Cagliani J Aziz M Tan C Brenner M Wang P . Extracellular cirp activates sting to exacerbate hemorrhagic shock. JCI Insight. (2021) 6:e143715. doi: 10.1172/jci.insight.143715, PMID: 34291735 PMC8410031

[59] Lin R Wang J Wu Y Yi Z Zhang Y Li L . Resolving neutrophils due to tram deletion renders protection against experimental sepsis. Inflammation Res. (2023) 72:1733–44. doi: 10.1007/s00011-023-01779-z, PMID: 37563334 PMC10727485

[60] Zhakeer G Zeng Y E G Maimaitiaili N Ju P Yao H . T(Reg) cells attenuate pulmonary venous remodeling in ph-lhd *via* nlrc3 signaling. Circ Res. (2025) 136:e113–e28. doi: 10.1161/circresaha.124.325201, PMID: 40235449

[61] Mettelman RC Allen EK Thomas PG . Mucosal immune responses to infection and vaccination in the respiratory tract. Immunity. (2022) 55:749–80. doi: 10.1016/j.immuni.2022.04.013, PMID: 35545027 PMC9087965

[62] Iwasaki A Medzhitov R . Control of adaptive immunity by the innate immune system. Nat Immunol. (2015) 16:343–53. doi: 10.1038/ni.3123, PMID: 25789684 PMC4507498

[63] Lugg ST Scott A Parekh D Naidu B Thickett DR . Cigarette smoke exposure and alveolar macrophages: mechanisms for lung disease. Thorax. (2022) 77:94–101. doi: 10.1136/thoraxjnl-2020-216296, PMID: 33986144 PMC8685655

[64] Li H Bradbury JA Edin ML Graves JP Gruzdev A Cheng J . Seh promotes macrophage phagocytosis and lung clearance of streptococcus pneumoniae. J Clin Invest. (2021) 131:e129679. doi: 10.1172/jci129679, PMID: 34591792 PMC8592545

[65] Deliyannis G Wong CY McQuilten HA Bachem A Clarke M Jia X . Tlr2-mediated activation of innate responses in the upper airways confers antiviral protection of the lungs. JCI Insight. (2021) 6:e140267. doi: 10.1172/jci.insight.140267, PMID: 33561017 PMC8021123

[66] Donovan ML Bielefeldt-Ohmann H Rollo RF McPherson SJ Schultz TE Mori G . Distinct contributions of the innate immune receptors tlr2 and rp105 to formation and architecture of structured lung granulomas in mice infected with mycobacterium tuberculosis. Immunology. (2023) 169:13–26. doi: 10.1111/imm.13606, PMID: 36370035

[67] Kang ZY Huang QY Zhen NX Xuan NX Zhou QC Zhao J . Heterogeneity of immune cells and their communications unveiled by transcriptome profiling in acute inflammatory lung injury. Front Immunol. (2024) 15:1382449. doi: 10.3389/fimmu.2024.1382449, PMID: 38745657 PMC11092984

[68] Pahari S Negi S Aqdas M Arnett E Schlesinger LS Agrewala JN . Induction of autophagy through clec4e in combination with tlr4: an innovative strategy to restrict the survival of mycobacterium tuberculosis. Autophagy. (2020) 16:1021–43. doi: 10.1080/15548627.2019.1658436, PMID: 31462144 PMC7469444

[69] Percier P De Prins S Tima G Beyaert R Grooten J Romano M . Aspergillusfumigatus recognition by dendritic cells negatively regulates allergic lung inflammation through a tlr2/myd88 pathway. Am J Respir Cell Mol Biol. (2021) 64:39–49. doi: 10.1165/rcmb.2020-0083OC, PMID: 32970964

[70] Fama A Midiri A Mancuso G Biondo C Lentini G Galbo R . Nucleic acid-sensing toll-like receptors play a dominant role in innate immune recognition of pneumococci. mBio. (2020) 11:e00415–20. doi: 10.1128/mBio.00415-20, PMID: 32209688 PMC7157524

[71] Grassin-Delyle S Abrial C Salvator H Brollo M Naline E Devillier P . The role of toll-like receptors in the production of cytokines by human lung macrophages. J Innate Immun. (2020) 12:63–73. doi: 10.1159/000494463, PMID: 30557876 PMC6959095

[72] Yin C Cheng L Pan J Chen L Xue Q Qin J . Regulatory role of gpr84 in the switch of alveolar macrophages from cd11b(Lo) to cd11b(Hi) status during lung injury process. Mucosal Immunol. (2020) 13:892–907. doi: 10.1038/s41385-020-0321-7, PMID: 32719411

[73] Liu X Boyer MA Holmgren AM Shin S . Legionella-infected macrophages engage the alveolar epithelium to metabolically reprogram myeloid cells and promote antibacterial inflammation. Cell Host Microbe. (2020) 28:683–98 e6. doi: 10.1016/j.chom.2020.07.019, PMID: 32841604 PMC9339267

[74] Jackson WD Giacomassi C Ward S Owen A Luis TC Spear S . Tlr7 activation at epithelial barriers promotes emergency myelopoiesis and lung antiviral immunity. Elife. (2023) 12:e85647. doi: 10.7554/eLife.85647, PMID: 37566453 PMC10465127

[75] Horn KJ Schopper MA Drigot ZG Clark SE . Airway prevotella promote tlr2-dependent neutrophil activation and rapid clearance of streptococcus pneumoniae from the lung. Nat Commun. (2022) 13:3321. doi: 10.1038/s41467-022-31074-0, PMID: 35680890 PMC9184549

[76] Girkin J Loo SL Esneau C Maltby S Mercuri F Chua B . Tlr2-mediated innate immune priming boosts lung anti-viral immunity. Eur Respir J. (2021) 58:2001584. doi: 10.1183/13993003.01584-2020, PMID: 33303547

[77] Wu JY Huang TW Hsieh YT Wang YF Yen CC Lee GL . Cancer-derived succinate promotes macrophage polarization and cancer metastasis *via* succinate receptor. Mol Cell. (2020) 77:213–27 e5. doi: 10.1016/j.molcel.2019.10.023, PMID: 31735641

[78] Han S Wang W Wang S Yang T Zhang G Wang D . Tumor microenvironment remodeling and tumor therapy based on M2-like tumor associated macrophage-targeting nano-complexes. Theranostics. (2021) 11:2892–916. doi: 10.7150/thno.50928, PMID: 33456579 PMC7806477

[79] Anfray C Varela CF Ummarino A Maeda A Sironi M Gandoy S . Polymeric nanocapsules loaded with poly(I:C) and resiquimod to reprogram tumor-associated macrophages for the treatment of solid tumors. Front Immunol. (2023) 14:1334800. doi: 10.3389/fimmu.2023.1334800, PMID: 38259462 PMC10800412

[80] Si J Ma Y Bi JW Xiong Y Lv C Li S . Shisa3 brakes resistance to egfr-tkis in lung adenocarcinoma by suppressing cancer stem cell properties. J Exp Clin Cancer Res. (2019) 38:481. doi: 10.1186/s13046-019-1486-3, PMID: 31801598 PMC6894286

[81] Zhang S Yu B Sheng C Yao C Liu Y Wang J . Shisa3 reprograms tumor-associated macrophages toward an antitumoral phenotype and enhances cancer immunotherapy. Adv Sci (Weinh). (2024) 11:e2403019. doi: 10.1002/advs.202403019, PMID: 39054639 PMC11423144

[82] Bolli E Scherger M Arnouk SM Pombo Antunes AR Strassburger D Urschbach M . Targeted repolarization of tumor-associated macrophages *via* imidazoquinoline-linked nanobodies. Adv Sci (Weinh). (2021) 8:2004574. doi: 10.1002/advs.202004574, PMID: 34026453 PMC8132149

[83] Palucka K Banchereau J . Cancer immunotherapy *via* dendritic cells. Nat Rev Cancer. (2012) 12:265–77. doi: 10.1038/nrc3258, PMID: 22437871 PMC3433802

[84] Xie D Han C Chen C Liao Z Campos de Souza S Niu Y . A scaffold vaccine to promote tumor antigen cross-presentation *via* sustained toll-like receptor-2 (Tlr2) activation. Bioact Mater. (2024) 37:315–30. doi: 10.1016/j.bioactmat.2024.03.035, PMID: 38694764 PMC11061615

[85] Wang J Guo B Sun Z Zhao S Cao L Zhong Z . Polymersomal poly(I:C) self-magnifies antitumor immunity by inducing immunogenic cell death and systemic immune activation. Adv Healthc Mater. (2024) 13:e2400784. doi: 10.1002/adhm.202400784, PMID: 38896790

[86] Sharma P Levy O Dowling DJ . The tlr5 agonist flagellin shapes phenotypical and functional activation of lung mucosal antigen presenting cells in neonatal mice. Front Immunol. (2020) 11:171. doi: 10.3389/fimmu.2020.00171, PMID: 32132997 PMC7039933

[87] Bosteels C Neyt K Vanheerswynghels M van Helden MJ Sichien D Debeuf N . Inflammatory type 2 cdcs acquire features of cdc1s and macrophages to orchestrate immunity to respiratory virus infection. Immunity. (2020) 52:1039–56.e9. doi: 10.1016/j.immuni.2020.04.005, PMID: 32392463 PMC7207120

[88] Zhang L Huang J Chen X Pan C He Y Su R . Self-assembly nanovaccine containing tlr7/8 agonist and stat3 inhibitor enhances tumor immunotherapy by augmenting tumor-specific immune response. J Immunother Cancer. (2021) 9:e003132. doi: 10.1136/jitc-2021-003132, PMID: 34452929 PMC8404452

[89] Fernandez-Rodriguez L Cianciaruso C Bill R Trefny MP Klar R Kirchhammer N . Dual tlr9 and pd-L1 targeting unleashes dendritic cells to induce durable antitumor immunity. J Immunother Cancer. (2023) 11:e006714. doi: 10.1136/jitc-2023-006714, PMID: 37208130 PMC10201251

[90] Liu L Mao Y Xu B Zhang X Fang C Ma Y . Induction of neutrophil extracellular traps during tissue injury: involvement of sting and toll-like receptor 9 pathways. Cell Prolif. (2019) 52:e12579. doi: 10.1111/cpr.12579, PMID: 30851061 PMC6536408

[91] Hagan RS Gomez JC Torres-Castillo J Martin JR Doerschuk CM . Tbk1 is required for host defense functions distinct from type I ifn expression and myeloid cell recruitment in murine streptococcus pneumoniae pneumonia. Am J Respir Cell Mol Biol. (2022) 66:671–81. doi: 10.1165/rcmb.2020-0311OC, PMID: 35358404 PMC9163639

[92] Tsai Z Carver KA Gong HH Kosai K Deng JC Worley MJ . Detailed mechanisms underlying neutrophil bactericidal activity against streptococcus pneumoniae. Biomedicines. (2023) 11:2252. doi: 10.3390/biomedicines11082252, PMID: 37626748 PMC10452576

[93] Matarazzo L Costa C Porte R Saliou JM Figeac M Delahaye F . Neutrophil subsets enhance the efficacy of host-directed therapy in pneumococcal pneumonia. Mucosal Immunol. (2024) 18:257–68. doi: 10.1016/j.mucimm.2024.11.009, PMID: 39592068

[94] Antoniak S Tatsumi K Schmedes CM Egnatz GJ Auriemma AC Bharathi V . Par1 regulation of cxcl1 expression and neutrophil recruitment to the lung in mice infected with influenza a virus. J Thromb Haemost. (2021) 19:1103–11. doi: 10.1111/jth.15221, PMID: 33346953 PMC8048419

[95] Liu R Zhu G Sun Y Li M Hu Z Cao P . Neutrophil infiltration associated genes on the prognosis and tumor immune microenvironment of lung adenocarcinoma. Front Immunol. (2023) 14:1304529. doi: 10.3389/fimmu.2023.1304529, PMID: 38204755 PMC10777728

[96] Lin Q Zong S Wang Y Zhou Y Wang K Shi F . Breast cancer-derived cav1 promotes lung metastasis by regulating integrin α6β4 and the recruitment and polarization of tumor-associated neutrophils. Int J Biol Sci. (2024) 20:5695–714. doi: 10.7150/ijbs.94153, PMID: 39494337 PMC11528463

[97] Zhang H Zhu X Friesen TJ Kwak JW Pisarenko T Mekvanich S . Annexin A2/tlr2/myd88 pathway induces arginase 1 expression in tumor-associated neutrophils. J Clin Invest. (2022) 132:e153643. doi: 10.1172/jci153643, PMID: 36377658 PMC9663166

[98] Cheng X Yu P Zhou X Zhu J Han Y Zhang C . Enhanced tumor homing of pathogen-mimicking liposomes driven by R848 stimulation: A new platform for synergistic oncology therapy. Acta Pharm Sin B. (2022) 12:924–38. doi: 10.1016/j.apsb.2021.08.018, PMID: 35256955 PMC8897206

[99] Hua Z Hou B . Tlr signaling in B-cell development and activation. Cell Mol Immunol. (2013) 10:103–6. doi: 10.1038/cmi.2012.61, PMID: 23241902 PMC4003046

[100] Reba SM Li Q Onwuzulike S Nagy N Fletcher S Parker K . Tlr2 on cd4+ and cd8+ T cells promotes control of mycobacterium tuberculosis infection. Eur J Immunol. (2024) 54:e2350715. doi: 10.1002/eji.202350715, PMID: 38446066

[101] Ashhurst AS Johansen MD Maxwell JWC Stockdale S Ashley CL Aggarwal A . Mucosal tlr2-activating protein-based vaccination induces potent pulmonary immunity and protection against sars-cov-2 in mice. Nat Commun. (2022) 13:6972. doi: 10.1038/s41467-022-34297-3, PMID: 36379950 PMC9665025

[102] Wørzner K Schmidt ST Zimmermann J Tami A Polacek C Fernandez-Antunez C . Intranasal recombinant protein subunit vaccine targeting tlr3 induces respiratory tract iga and cd8 T cell responses and protects against respiratory virus infection. EBioMedicine. (2025) 113:105615. doi: 10.1016/j.ebiom.2025.105615, PMID: 39983329 PMC11893338

[103] Ko KH Bae HS Park JW Lee JS Park S Heo J . A vaccine platform targeting lung-resident memory cd4(+) T-cells provides protection against heterosubtypic influenza infections in mice and ferrets. Nat Commun. (2024) 15:10368. doi: 10.1038/s41467-024-54620-4, PMID: 39609429 PMC11604757

[104] Do KTH Willenzon S Ristenpart J Janssen A Volz A Sutter G . The effect of toll-like receptor agonists on the immunogenicity of mva-sars-2-S vaccine after intranasal administration in mice. Front Cell Infect Microbiol. (2023) 13:1259822. doi: 10.3389/fcimb.2023.1259822, PMID: 37854858 PMC10580083

[105] Holland T Wohlleber D Marx S Kreutzberg T Vento-Asturias S Schmitt-Mbamunyo C . Rescue of T-cell function during persistent pulmonary adenoviral infection by toll-like receptor 9 activation. J Allergy Clin Immunol. (2018) 141:416–9.e10. doi: 10.1016/j.jaci.2017.06.048, PMID: 28826775

[106] Yan W Zhao YS Xie K Xing Y Xu F . Aspergillus fumigatus influences gasdermin-D-dependent pyroptosis of the lung *via* regulating toll-like receptor 2-mediated regulatory T cell differentiation. J Immunol Res. (2021) 2021:5538612. doi: 10.1155/2021/5538612, PMID: 34222495 PMC8219420

[107] Jannuzzi GP de Almeida JRF Amarante-Mendes GP Romera LMD Kaihami GH Vasconcelos JR . Tlr3 is a negative regulator of immune responses against paracoccidioides brasiliensis. Front Cell Infect Microbiol. (2018) 8:426. doi: 10.3389/fcimb.2018.00426, PMID: 30687643 PMC6335947

[108] Kaminski VL Borges BM Santos BV Preite NW Calich VLG Loures FV . Mdscs use a complex molecular network to suppress T-cell immunity in a pulmonary model of fungal infection. Front Cell Infect Microbiol. (2024) 14:1392744. doi: 10.3389/fcimb.2024.1392744, PMID: 39035356 PMC11257977

[109] Wilson AS Randall KL Pettitt JA Ellyard JI Blumenthal A Enders A . Neutrophil extracellular traps and their histones promote th17 cell differentiation directly *via* tlr2. Nat Commun. (2022) 13:528. doi: 10.1038/s41467-022-28172-4, PMID: 35082281 PMC8792063

[110] Choi HG Kim WS Back YW Kim H Kwon KW Kim JS . Mycobacterium tuberculosis rpfe promotes simultaneous th1- and th17-type T-cell immunity *via* tlr4-dependent maturation of dendritic cells. Eur J Immunol. (2015) 45:1957–71. doi: 10.1002/eji.201445329, PMID: 25907170

[111] Marinaik CB Kingstad-Bakke B Lee W Hatta M Sonsalla M Larsen A . Programming multifaceted pulmonary T cell immunity by combination adjuvants. Cell Rep Med. (2020) 1:100095. doi: 10.1016/j.xcrm.2020.100095, PMID: 32984856 PMC7508055

[112] Exposito F Redrado M Houry M Hastings K Molero-Abraham M Lozano T . Pten loss confers resistance to anti-pd-1 therapy in non-small cell lung cancer by increasing tumor infiltration of regulatory T cells. Cancer Res. (2023) 83:2513–26. doi: 10.1158/0008-5472.Can-22-3023, PMID: 37311042

[113] Pfirschke C Engblom C Rickelt S Cortez-Retamozo V Garris C Pucci F . Immunogenic chemotherapy sensitizes tumors to checkpoint blockade therapy. Immunity. (2016) 44:343–54. doi: 10.1016/j.immuni.2015.11.024, PMID: 26872698 PMC4758865

[114] Yao Y Li J Qu K Wang Y Wang Z Lu W . Immunotherapy for lung cancer combining the oligodeoxynucleotides of tlr9 agonist and tgf-β2 inhibitor. Cancer Immunol Immunother. (2023) 72:1103–20. doi: 10.1007/s00262-022-03315-0, PMID: 36326892 PMC10992143

[115] Farahnak K Bai YZ Yokoyama Y Morkan DB Liu Z Amrute JM . B cells mediate lung ischemia/reperfusion injury by recruiting classical monocytes via synergistic B cell receptor/tlr4 signaling. J Clin Invest. (2024) 134:e170118. doi: 10.1172/JCI170118, PMID: 38488011 PMC10940088

[116] Pei J Ding X Fan Y Rice-Ficht A Ficht TA . Toll-like receptors are critical for clearance of brucella and play different roles in development of adaptive immunity following aerosol challenge in mice. Front Cell Infect Microbiol. (2012) 2:115. doi: 10.3389/fcimb.2012.00115, PMID: 22973560 PMC3435510

[117] Wang C Khatun MS Ellsworth CR Chen Z Islamuddin M Nisperuza Vidal AK . Deficiency of tlr7 and irf7 in mice increases the severity of covid-19 through the reduced interferon production. Commun Biol. (2024) 7:1162. doi: 10.1038/s42003-024-06872-5, PMID: 39289468 PMC11408513

[118] Li C To KKW Zhang AJX Lee ACY Zhu H Mak WWN . Co-stimulation with tlr7 agonist imiquimod and inactivated influenza virus particles promotes mouse B cell activation, differentiation, and accelerated antigen specific antibody production. Front Immunol. (2018) 9:2370. doi: 10.3389/fimmu.2018.02370, PMID: 30369932 PMC6194170

[119] Akkaya M Traba J Roesler AS Miozzo P Akkaya B Theall BP . Second signals rescue B cells from activation-induced mitochondrial dysfunction and death. Nat Immunol. (2018) 19:871–84. doi: 10.1038/s41590-018-0156-5, PMID: 29988090 PMC6202187

[120] Pawar RD Ramanjaneyulu A Kulkarni OP Lech M Segerer S Anders HJ . Inhibition of toll-like receptor-7 (Tlr-7) or tlr-7 plus tlr-9 attenuates glomerulonephritis and lung injury in experimental lupus. J Am Soc Nephrol. (2007) 18:1721–31. doi: 10.1681/asn.2006101162, PMID: 17460144

[121] Yoshizaki A Taniguchi T Saigusa R Fukasawa T Ebata S Numajiri H . Nucleosome in patients with systemic sclerosis: possible association with immunological abnormalities *via* abnormal activation of T and B cells. Ann Rheum Dis. (2016) 75:1858–65. doi: 10.1136/annrheumdis-2015-207405, PMID: 26567180

[122] Mavropoulos A Simopoulou T Varna A Liaskos C Katsiari CG Bogdanos DP . Breg cells are numerically decreased and functionally impaired in patients with systemic sclerosis. Arthritis Rheumatol. (2016) 68:494–504. doi: 10.1002/art.39437, PMID: 26414243

[123] Yoshizaki A Iwata Y Komura K Ogawa F Hara T Muroi E . Cd19 regulates skin and lung fibrosis *via* toll-like receptor signaling in a model of bleomycin-induced scleroderma. Am J Pathol. (2008) 172:1650–63. doi: 10.2353/ajpath.2008.071049, PMID: 18467694 PMC2408424

[124] Kato A Truong-Tran AQ Scott AL Matsumoto K Schleimer RP . Airway epithelial cells produce B cell-activating factor of tnf family by an ifn-beta-dependent mechanism. J Immunol. (2006) 177:7164–72. doi: 10.4049/jimmunol.177.10.7164, PMID: 17082634 PMC2804942

[125] Wang C Oishi K Kobayashi T Fujii K Horii M Fushida N . The role of tlr7 and tlr9 in the pathogenesis of systemic sclerosis. Int J Mol Sci. (2024) 25:6133. doi: 10.3390/ijms25116133, PMID: 38892317 PMC11172923

[126] Kugler-Umana O Zhang W Kuang Y Liang J Castonguay CH Tonkonogy SL . Igd(+) age-associated B cells are the progenitors of the main T-independent B cell response to infection that generates protective ab and can be induced by an inactivated vaccine in the aged. Aging Cell. (2022) 21:e13705. doi: 10.1111/acel.13705, PMID: 36056604 PMC9577953

[127] van der Vlugt L Obieglo K Ozir-Fazalalikhan A Sparwasser T Haeberlein S Smits HH . Schistosome-induced pulmonary B cells inhibit allergic airway inflammation and display a reduced th2-driving function. Int J Parasitol. (2017) 47:545–54. doi: 10.1016/j.ijpara.2017.02.002, PMID: 28385494

[128] Lv Y Kim K Sheng Y Cho J Qian Z Zhao YY . Yap controls endothelial activation and vascular inflammation through traf6. Circ Res. (2018) 123:43–56. doi: 10.1161/circresaha.118.313143, PMID: 29794022 PMC6014930

[129] Renaud L da Silveira WA Takamura N Hardiman G Feghali-Bostwick C . Prominence of il6, igf, tlr, and bioenergetics pathway perturbation in lung tissues of scleroderma patients with pulmonary fibrosis. Front Immunol. (2020) 11:383. doi: 10.3389/fimmu.2020.00383, PMID: 32210969 PMC7075854

[130] Zhu S Yu Y Qu M Qiu Z Zhang H Miao C . Neutrophil extracellular traps contribute to immunothrombosis formation *via* the sting pathway in sepsis-associated lung injury. Cell Death Discov. (2023) 9:315. doi: 10.1038/s41420-023-01614-8, PMID: 37626060 PMC10457383

[131] Xu L Hu W Zhang J Qu J . Knockdown of Versican 1 in Lung Fibroblasts Aggravates Lipopolysaccharide-Induced Acute Inflammation through up-Regulation of the Sp1-Toll-Like Receptor 2-Nf-κb Axis: A Potential Barrier to Promising Versican-Targeted Therapy. Int Immunopharmacol. (2023) 121:110406. doi: 10.1016/j.intimp.2023.110406, PMID: 37311354

[132] Wu Y Yu X Wang Y Huang Y Tang J Gong S . Ruscogenin alleviates lps-triggered pulmonary endothelial barrier dysfunction through targeting nmmhc iia to modulate tlr4 signaling. Acta Pharm Sin B. (2022) 12:1198–212. doi: 10.1016/j.apsb.2021.09.017, PMID: 35530141 PMC9069402

[133] Huang H Zhu J Gu L Hu J Feng X Huang W . Tlr7 mediates acute respiratory distress syndrome in sepsis by sensing extracellular mir-146a. Am J Respir Cell Mol Biol. (2022) 67:375–88. doi: 10.1165/rcmb.2021-0551OC, PMID: 35679261 PMC9447138

[134] Yeh FC Chen CN Xie CY Baxan N Zhao L Ashek A . Tlr7/8 activation induces autoimmune vasculopathy and causes severe pulmonary arterial hypertension. Eur Respir J. (2023) 62:2300204. doi: 10.1183/13993003.00204-2023, PMID: 37290790 PMC10356963

[135] Wedgwood S Gerard K Halloran K Hanhauser A Monacelli S Warford C . Intestinal dysbiosis and the developing lung: the role of toll-like receptor 4 in the gut-lung axis. Front Immunol. (2020) 11:357. doi: 10.3389/fimmu.2020.00357, PMID: 32194566 PMC7066082

[136] Cheng X Jiang W Chen Y Zou B Wang Z Gan L . Acyloxyacyl hydrolase promotes pulmonary defense by preventing alveolar macrophage tolerance. PloS Pathog. (2023) 19:e1011556. doi: 10.1371/journal.ppat.1011556, PMID: 37498977 PMC10409266

[137] Torres A Cilloniz C Niederman MS Menendez R Chalmers JD Wunderink RG . Pneumonia. Nat Rev Dis Primers. (2021) 7:25. doi: 10.1038/s41572-021-00259-0, PMID: 33833230

[138] Chen T Chen C Zhang Z Zou Y Peng M Wang Y . Toll-like receptor 4 knockout ameliorates neuroinflammation due to lung-brain interaction in mechanically ventilated mice. Brain Behav Immun. (2016) 56:42–55. doi: 10.1016/j.bbi.2016.04.004, PMID: 27067748

[139] Takahashi M Chen-Yoshikawa TF Menju T Ohata K Kondo T Motoyama H . Inhibition of toll-like receptor 4 signaling ameliorates lung ischemia-reperfusion injury in acute hyperglycemic conditions. J Heart Lung Transplant. (2016) 35:815–22. doi: 10.1016/j.healun.2015.12.032, PMID: 26922276

[140] Strassheim D Kim JY Park JS Mitra S Abraham E . Involvement of ship in tlr2-induced neutrophil activation and acute lung injury. J Immunol. (2005) 174:8064–71. doi: 10.4049/jimmunol.174.12.8064, PMID: 15944314

[141] Zhang W Zhou H Jiang Y He J Yao Y Wang J . Acinetobacter baumannii outer membrane protein a induces pulmonary epithelial barrier dysfunction and bacterial translocation through the tlr2/iqgap1 axis. Front Immunol. (2022) 13:927955. doi: 10.3389/fimmu.2022.927955, PMID: 35844614 PMC9280087

[142] Tang J Xu L Zeng Y Gong F . Effect of gut microbiota on lps-induced acute lung injury by regulating the tlr4/nf-kb signaling pathway. Int Immunopharmacol. (2021) 91:107272. doi: 10.1016/j.intimp.2020.107272, PMID: 33360370

[143] Sayers I Thakker D Billington C Kreideweiss S Grundl MA Bouyssou T . Interleukin-1 receptor-associated kinase 4 (Irak4) is a critical regulator of inflammatory signalling through toll-like receptors 4 and 7/8 in murine and human lungs. Br J Pharmacol. (2024) 181:4647–57. doi: 10.1111/bph.16509, PMID: 39137914 PMC7618454

[144] Liu PY Chen CY Lin YL Lin CM Tsai WC Tsai YL . Rnf128 regulates neutrophil infiltration and myeloperoxidase functions to prevent acute lung injury. Cell Death Dis. (2023) 14:369. doi: 10.1038/s41419-023-05890-1, PMID: 37344492 PMC10284794

[145] Dai F Zhang X Ma G Li W . Acod1 mediates staphylococcus aureus-induced inflammatory response *via* the tlr4/nf-kappab signaling pathway. Int Immunopharmacol. (2024) 140:112924. doi: 10.1016/j.intimp.2024.112924, PMID: 39133958

[146] He J Yuan R Cui X Cui Y Han S Wang QQ . Anemoside B4 protects against klebsiella pneumoniae- and influenza virus fm1-induced pneumonia *via* the tlr4/myd88 signaling pathway in mice. Chin Med. (2020) 15:68. doi: 10.1186/s13020-020-00350-w, PMID: 32625244 PMC7330533

[147] Casilag F Matarazzo L Franck S Figeac M Michelet R Kloft C . The biosynthetic monophosphoryl lipid a enhances the therapeutic outcome of antibiotic therapy in pneumococcal pneumonia. ACS Infect Dis. (2021) 7:2164–75. doi: 10.1021/acsinfecdis.1c00176, PMID: 34260199

[148] Park J Kim S Lim H Liu A Hu S Lee J . Therapeutic effects of human mesenchymal stem cell microvesicles in an ex vivo perfused human lung injured with severe E. Coli Pneumonia. Thorax. (2019) 74:43–50. doi: 10.1136/thoraxjnl-2018-211576, PMID: 30076187 PMC6295323

[149] López-Gálvez R Fleurot I Chamero P Trapp S Olivier M Chevaleyre C . Airway administration of flagellin regulates the inflammatory response to pseudomonas aeruginosa. Am J Respir Cell Mol Biol. (2021) 65:378–89. doi: 10.1165/rcmb.2021-0125OC, PMID: 34102087 PMC8525202

[150] Baldry M Costa C Zeroual Y Cayet D Pardessus J Soulard D . Targeted delivery of flagellin by nebulization offers optimized respiratory immunity and defense against pneumococcal pneumonia. Antimicrob Agents Chemother. (2024) 68:e0086624. doi: 10.1128/aac.00866-24, PMID: 39480071 PMC11619323

[151] Xu H Huang L Luo Q Tu Q Liu J Yu R . Absence of toll-like receptor 7 protects mice against pseudomonas aeruginosa pneumonia. Int Immunopharmacol. (2021) 96:107739. doi: 10.1016/j.intimp.2021.107739, PMID: 33984723

[152] Sekheri M El Kebir D Edner N Filep JG . 15-epi-lxa(4) and 17-epi-rvd1 restore tlr9-mediated impaired neutrophil phagocytosis and accelerate resolution of lung inflammation. Proc Natl Acad Sci U.S.A. (2020) 117:7971–80. doi: 10.1073/pnas.1920193117, PMID: 32205444 PMC7149425

[153] Bierwagen J Wiegand M Laakmann K Danov O Limburg H Herbel SM . Bacterial vesicles block viral replication in macrophages *via* tlr4-trif-axis. Cell Commun Signal. (2023) 21:65. doi: 10.1186/s12964-023-01086-4, PMID: 36978183 PMC10045439

[154] Zhao G Gentile ME Xue L Cosgriff CV Weiner AI Adams-Tzivelekidis S . Vascular endothelial-derived sparcl1 exacerbates viral pneumonia through pro-inflammatory macrophage activation. Nat Commun. (2024) 15:4235. doi: 10.1038/s41467-024-48589-3, PMID: 38762489 PMC11102455

[155] Meidert AS Hermann S Brandes F Kirchner B Buschmann D Billaud JN . Extracellular vesicle associated mirnas regulate signaling pathways involved in covid-19 pneumonia and the progression to severe acute respiratory corona virus-2 syndrome. Front Immunol. (2021) 12:784028. doi: 10.3389/fimmu.2021.784028, PMID: 34956213 PMC8696174

[156] Broggi A Ghosh S Sposito B Spreafico R Balzarini F Lo Cascio A . Type iii interferons disrupt the lung epithelial barrier upon viral recognition. Science. (2020) 369:706–12. doi: 10.1126/science.abc3545, PMID: 32527925 PMC7292499

[157] Ghimire R Shrestha R Amaradhi R Liu L More S Ganesh T . Toll-like receptor 7 (Tlr7)-mediated antiviral response protects mice from lethal sars-cov-2 infection. J Virol. (2025) 99:e0166824. doi: 10.1128/jvi.01668-24, PMID: 40162785 PMC12090760

[158] Miles MA Liong S Liong F Trollope GS Wang H Brooks RD . Tlr7 promotes acute inflammatory-driven lung dysfunction in influenza-infected mice but prevents late airway hyperresponsiveness. Int J Mol Sci. (2024) 25:13699. doi: 10.3390/ijms252413699, PMID: 39769461 PMC11678220

[159] Kim J Yuan Y Agaronyan K Zhao A Wang VD Gau D . Damage sensing through tlr9 regulates inflammatory and antiviral responses during influenza infection. Mucosal Immunol. (2025) 18:537–48. doi: 10.1016/j.mucimm.2025.01.008, PMID: 39884393 PMC12205908

[160] Pantaleón García J Wurster S Albert ND Bharadwaj U Bhoda K Kulkarni VK . Immunotherapy with nebulized pattern recognition receptor agonists restores severe immune paralysis and improves outcomes in mice with influenza-associated pulmonary aspergillosis. mBio. (2025) 16:e0406124. doi: 10.1128/mbio.04061-24, PMID: 40197039 PMC12077147

[161] Ling LJ Lu Y Zhang YY Zhu HY Tu P Li H . Flavonoids from houttuynia cordata attenuate H1n1-induced acute lung injury in mice *via* inhibition of influenza virus and toll-like receptor signalling. Phytomedicine. (2020) 67:153150. doi: 10.1016/j.phymed.2019.153150, PMID: 31958713

[162] Dang EV Lei S Radkov A Volk RF Zaro BW Madhani HD . Secreted fungal virulence effector triggers allergic inflammation *via* tlr4. Nature. (2022) 608:161–7. doi: 10.1038/s41586-022-05005-4, PMID: 35896747 PMC9744105

[163] Luo H He J Qin L Chen Y Chen L Li R . Mycoplasma pneumoniae lipids license tlr-4 for activation of nlrp3 inflammasome and autophagy to evoke a proinflammatory response. Clin Exp Immunol. (2021) 203:66–79. doi: 10.1111/cei.13510, PMID: 32894580 PMC7744503

[164] Tamiya S Yoshikawa E Ogura M Kuroda E Suzuki K Yoshioka Y . Vaccination using inactivated mycoplasma pneumoniae induces detrimental infiltration of neutrophils after subsequent infection in mice. Vaccine. (2020) 38:4979–87. doi: 10.1016/j.vaccine.2020.05.074, PMID: 32536549

[165] Miller RL Grayson MH Strothman K . Advances in asthma: new understandings of asthma's natural history, risk factors, underlying mechanisms, and clinical management. J Allergy Clin Immunol. (2021) 148:1430–41. doi: 10.1016/j.jaci.2021.10.001, PMID: 34655640

[166] Venkatesan P . Gold copd report: 2024 update. Lancet Respir Med. (2024) 12:15–6. doi: 10.1016/S2213-2600(23)00461-7, PMID: 38061380

[167] Sukkar MB Xie S Khorasani NM Kon OM Stanbridge R Issa R . Toll-like receptor 2, 3, and 4 expression and function in human airway smooth muscle. J Allergy Clin Immunol. (2006) 118:641–8. doi: 10.1016/j.jaci.2006.05.013, PMID: 16950283

[168] Park MK Park HK Yu HS . Toll-like receptor 2 mediates acanthamoeba-induced allergic airway inflammatory response in mice. PloS Negl Trop Dis. (2023) 17:e0011085. doi: 10.1371/journal.pntd.0011085, PMID: 36706056 PMC9882781

[169] Wu Y Gou Y Wang T Li P Li Y Lu X . Exportin xpo6 upregulation activates the tlr2/myd88/nf-kappab signaling by facilitating tlr2 mrna nuclear export in copd pulmonary monocytes. Int Immunopharmacol. (2024) 135:112310. doi: 10.1016/j.intimp.2024.112310, PMID: 38788453

[170] Lv J Yu Q Lv J Di C Lin X Su W . Airway epithelial tslp production of tlr2 drives type 2 immunity in allergic airway inflammation. Eur J Immunol. (2018) 48:1838–50. doi: 10.1002/eji.201847663, PMID: 30184256 PMC6282509

[171] Wu YF Li ZY Dong LL Li WJ Wu YP Wang J . Inactivation of mtor promotes autophagy-mediated epithelial injury in particulate matter-induced airway inflammation. Autophagy. (2020) 16:435–50. doi: 10.1080/15548627.2019.1628536, PMID: 31203721 PMC6999647

[172] Liu G Haw TJ Starkey MR Philp AM Pavlidis S Nalkurthi C . Tlr7 promotes smoke-induced experimental lung damage through the activity of mast cell tryptase. Nat Commun. (2023) 14:7349. doi: 10.1038/s41467-023-42913-z, PMID: 37963864 PMC10646046

[173] Jha A Thwaites RS Tunstall T Kon OM Shattock RJ Hansel TT . Increased nasal mucosal interferon and ccl13 response to a tlr7/8 agonist in asthma and allergic rhinitis. J Allergy Clin Immunol. (2021) 147:694–703.e12. doi: 10.1016/j.jaci.2020.07.012, PMID: 32717253

[174] Wang X Wu K Keeler SP Mao D Agapov EV Zhang Y . Tlr3-activated monocyte-derived dendritic cells trigger progression from acute viral infection to chronic disease in the lung. J Immunol. (2021) 206:1297–314. doi: 10.4049/jimmunol.2000965, PMID: 33514511 PMC7946811

[175] Tei R Iijima K Matsumoto K Kobayashi T Lama J Jacobsen EA . Tlr3-driven ifn-beta antagonizes stat5-activating cytokines and suppresses innate type 2 response in the lung. J Allergy Clin Immunol. (2022) 149:1044–59 e5. doi: 10.1016/j.jaci.2021.07.041, PMID: 34428519 PMC8859010

[176] Chen J Wang T Li X Gao L Wang K Cheng M . DNA of neutrophil extracellular traps promote nf-κb-dependent autoimmunity *via* cgas/tlr9 in chronic obstructive pulmonary disease. Signal Transduct Target Ther. (2024) 9:163. doi: 10.1038/s41392-024-01881-6, PMID: 38880789 PMC11180664

[177] Jansen K Cevhertas L Ma S Satitsuksanoa P Akdis M van de Veen W . Regulatory B cells, a to Z. Allergy. (2021) 76:2699–715. doi: 10.1111/all.14763, PMID: 33544905

[178] Whitehead GS Hussain S Fannin R Trempus CS Innes CL Schurman SH . Tlr5 activation exacerbates airway inflammation in asthma. Lung. (2020) 198:289–98. doi: 10.1007/s00408-020-00337-2, PMID: 32060608 PMC7123460

[179] Wilson RH Maruoka S Whitehead GS Foley JF Flake GP Sever ML . The toll-like receptor 5 ligand flagellin promotes asthma by priming allergic responses to indoor allergens. Nat Med. (2012) 18:1705–10. doi: 10.1038/nm.2920, PMID: 23064463 PMC3493750

[180] Shim JU Lee SE Hwang W Lee C Park JW Sohn JH . Flagellin suppresses experimental asthma by generating regulatory dendritic cells and T cells. J Allergy Clin Immunol. (2016) 137:426–35. doi: 10.1016/j.jaci.2015.07.010, PMID: 26303344

[181] Maher TM . Interstitial lung disease: A review. Jama. (2024) 331:1655–65. doi: 10.1001/jama.2024.3669, PMID: 38648021

[182] Moss BJ Ryter SW Rosas IO . Pathogenic mechanisms underlying idiopathic pulmonary fibrosis. Annu Rev Pathol. (2022) 17:515–46. doi: 10.1146/annurev-pathol-042320-030240, PMID: 34813355

[183] McElroy AN Invernizzi R Laskowska JW O'Neill A Doroudian M Moghoofei M . Candidate role for toll-like receptor 3 L412f polymorphism and infection in acute exacerbation of idiopathic pulmonary fibrosis. Am J Respir Crit Care Med. (2022) 205:550–62. doi: 10.1164/rccm.202010-3880OC, PMID: 34985402 PMC12042197

[184] Trujillo G Regueiro-Ren A Liu C Hu B Sun Y Ahangari F . Toll-like receptor 9 inhibition mitigates fibroproliferative responses in translational models of pulmonary fibrosis. Am J Respir Crit Care Med. (2024) 211:91–102. doi: 10.1164/rccm.202401-0065OC, PMID: 39189851 PMC11755360

[185] Carter H Costa RM Adams TS Gilchrist TM Emch CE Bame M . Cd103+ Dendritic cell-fibroblast crosstalk *via* tlr9, tdo2, and ahr signaling drives lung fibrogenesis. JCI Insight. (2025) 10:e177072. doi: 10.1172/jci.insight.177072, PMID: 39964756 PMC11949071

[186] Ren C Wang Q Fan S Mi T Zhang Z He D . Toll-like receptor 9 aggravates pulmonary fibrosis by promoting nlrp3-mediated pyroptosis of alveolar epithelial cells. Inflammation. (2024) 47:1744–61. doi: 10.1007/s10753-024-02006-5, PMID: 38498270

[187] Yang D Chen X Wang J Lou Q Lou Y Li L . Dysregulated lung commensal bacteria drive interleukin-17b production to promote pulmonary fibrosis through their outer membrane vesicles. Immunity. (2019) 50:692–706.e7. doi: 10.1016/j.immuni.2019.02.001, PMID: 30824326

[188] Li S Liu G Gu M Li Y Li Y Ji Z . A novel therapeutic approach for ipf: based on the "Autophagy - apoptosis" Balance regulation of zukamu granules in alveolar macrophages. J Ethnopharmacol. (2022) 297:115568. doi: 10.1016/j.jep.2022.115568, PMID: 35868548

[189] Long L Dai X Yao T Zhang X Jiang G Cheng X . Mefunidone alleviates silica-induced inflammation and fibrosis by inhibiting the tlr4-nf-κb/mapk pathway and attenuating pyroptosis in murine macrophages. BioMed Pharmacother. (2024) 178:117216. doi: 10.1016/j.biopha.2024.117216, PMID: 39096618

[190] Hu Y Yang L Huang L Zeng C Ren S . M6a reader igf2bp1 facilitates macrophage glycolytic metabolism and fibrotic phenotype by stabilizing thbs1 mrna to promote pulmonary fibrosis. Cell Mol Life Sci. (2025) 82:157. doi: 10.1007/s00018-025-05673-1, PMID: 40220148 PMC11993514

[191] Spagnolo P Distler O Ryerson CJ Tzouvelekis A Lee JS Bonella F . Mechanisms of progressive fibrosis in connective tissue disease (Ctd)-associated interstitial lung diseases (Ilds). Ann Rheum Dis. (2021) 80:143–50. doi: 10.1136/annrheumdis-2020-217230, PMID: 33037004 PMC7815631

[192] Ryu C Walia A Ortiz V Perry C Woo S Reeves BC . Bioactive plasma mitochondrial DNA is associated with disease progression in scleroderma-associated interstitial lung disease. Arthritis Rheumatol. (2020) 72:1905–15. doi: 10.1002/art.41418, PMID: 32602227 PMC8081728

[193] Ehlers C Thiele T Biermann H Traidl S Bruns L Ziegler A . Toll-like receptor 8 is expressed in monocytes in contrast to plasmacytoid dendritic cells and mediates aberrant interleukin-10 responses in patients with systemic sclerosis. Arthritis Rheumatol. (2025) 77:59–66. doi: 10.1002/art.42964, PMID: 39112920 PMC11685002

[194] van Bon L Affandi AJ Broen J Christmann RB Marijnissen RJ Stawski L . Proteome-wide analysis and cxcl4 as a biomarker in systemic sclerosis. N Engl J Med. (2014) 370:433–43. doi: 10.1056/NEJMoa1114576, PMID: 24350901 PMC4040466

[195] Bhattacharyya S Wang W Tamaki Z Shi B Yeldandi A Tsukimi Y . Pharmacological inhibition of toll-like receptor-4 signaling by tak242 prevents and induces regression of experimental organ fibrosis. Front Immunol. (2018) 9:2434. doi: 10.3389/fimmu.2018.02434, PMID: 30405628 PMC6207051

[196] Gan YZ Zhang LH Ma L Sun F Li YH An Y . Risk factors of interstitial lung diseases in clinically amyopathic dermatomyositis. Chin Med J (Engl). (2020) 133:644–9. doi: 10.1097/CM9.0000000000000691, PMID: 32049748 PMC7190228

[197] Ichimura Y Konishi R Shobo M Tanaka R Kubota N Kayama H . Autoimmunity against melanoma differentiation-associated gene 5 induces interstitial lung disease mimicking dermatomyositis in mice. Proc Natl Acad Sci U.S.A. (2024) 121:e2313070121. doi: 10.1073/pnas.2313070121, PMID: 38588434 PMC11032490

[198] Sun WC Sun YC Lin H Yan B Shi GX . Dysregulation of the type I interferon system in adult-onset clinically amyopathic dermatomyositis has a potential contribution to the development of interstitial lung disease. Br J Dermatol. (2012) 167:1236–44. doi: 10.1111/j.1365-2133.2012.11145.x, PMID: 23013528

[199] Xu W Huang M Dong R Yan S An Y Liu B . Anti-carbamylated protein antibodies drive aec ii toward a profibrotic phenotype by interacting with carbamylated tlr5. Rheumatol (Oxford). (2024) 63:2874–86. doi: 10.1093/rheumatology/keae111, PMID: 38366924

[200] Hooftman A Peace CG Ryan DG Day EA Yang M McGettrick AF . Macrophage fumarate hydratase restrains mtrna-mediated interferon production. Nature. (2023) 615:490–8. doi: 10.1038/s41586-023-05720-6, PMID: 36890227 PMC10411300

[201] Rai P Janardhan KS Meacham J Madenspacher JH Lin WC Karmaus PWF . Irgm1 links mitochondrial quality control to autoimmunity. Nat Immunol. (2021) 22:312–21. doi: 10.1038/s41590-020-00859-0, PMID: 33510463 PMC7906953

[202] Wu J Singh K Lin A Meadows AM Wu K Shing V . Boosting nad+ Blunts tlr4-induced type I ifn in control and systemic lupus erythematosus monocytes. J Clin Invest. (2022) 132:e139828. doi: 10.1172/jci139828, PMID: 35025762 PMC8884917

[203] Hsieh YT Chen YC Chou YC Kuo PY Yen YT Tsai HW . Long noncoding rna snhg16 regulates tlr4-mediated autophagy and netosis formation in alveolar hemorrhage associated with systemic lupus erythematosus. J BioMed Sci. (2023) 30:78. doi: 10.1186/s12929-023-00969-5, PMID: 37700342 PMC10496234

[204] Peng D Li J Li Y Bai L Xiong A He X . Mmp14(High) macrophages orchestrate progressive pulmonary fibrosis in sr-ag-induced hypersensitivity pneumonitis. Pharmacol Res. (2024) 200:107070. doi: 10.1016/j.phrs.2024.107070, PMID: 38218353

[205] Daito H Kikuchi T Sakakibara T Gomi K Damayanti T Zaini J . Mycobacterial hypersensitivity pneumonitis requires tlr9-myd88 in lung cd11b+ Cd11c+ Cells. Eur Respir J. (2011) 38:688–701. doi: 10.1183/09031936.00177110, PMID: 21273385

[206] Cheng D Lian W Jia X Wang T Sun W Jia Z . Senescent endothelial cell-derived galectin 3 promotes silicosis through endothelial-fibroblast and endothelial-macrophage crosstalk. J Hazard Mater. (2025) 489:137605. doi: 10.1016/j.jhazmat.2025.137605, PMID: 39955992

[207] Jia Q Wang H Wang Y Xue W Jiang Q Wang J . Investigation of the mechanism of silica-induced pulmonary fibrosis: the role of lung microbiota dysbiosis and the lps/tlr4 signaling pathway. Sci Total Environ. (2024) 912:168948. doi: 10.1016/j.scitotenv.2023.168948, PMID: 38048996

[208] Kim MJ Kim JY Shin JH Kang Y Lee JS Son J . Ffar2 antagonizes tlr2- and tlr3-induced lung cancer progression *via* the inhibition of ampk-tak1 signaling axis for the activation of nf-kappab. Cell Biosci. (2023) 13:102. doi: 10.1186/s13578-023-01038-y, PMID: 37287005 PMC10249240

[209] Millar FR Pennycuick A Muir M Quintanilla A Hari P Freyer E . Toll-like receptor 2 orchestrates a tumor suppressor response in non-small cell lung cancer. Cell Rep. (2022) 41:111596. doi: 10.1016/j.celrep.2022.111596, PMID: 36351380 PMC10197427

[210] Chen J Sun W Zhang H Ma J Xu P Yu Y . Macrophages reprogrammed by lung cancer microparticles promote tumor development *via* release of il-1β. Cell Mol Immunol. (2020) 17:1233–44. doi: 10.1038/s41423-019-0313-2, PMID: 31649305 PMC7784894

[211] Debnath J Gammoh N Ryan KM . Autophagy and autophagy-related pathways in cancer. Nat Rev Mol Cell Biol. (2023) 24:560–75. doi: 10.1038/s41580-023-00585-z, PMID: 36864290 PMC9980873

[212] Kim MJ Lee JS Kim JY Choi B Son J Min Y . Crbn is downregulated in lung cancer and negatively regulates tlr2, 4 and 7 stimulation in lung cancer cells. Clin Transl Med. (2022) 12:e1050. doi: 10.1002/ctm2.1050, PMID: 36164994 PMC9513676

[213] McGinnis CS Miao Z Superville D Yao W Goga A Reticker-Flynn NE . The temporal progression of lung immune remodeling during breast cancer metastasis. Cancer Cell. (2024) 42:1018–31.e6. doi: 10.1016/j.ccell.2024.05.004, PMID: 38821060 PMC11255555

[214] Kim MJ Min Y Jeong SK Son J Kim JY Lee JS . Usp15 negatively regulates lung cancer progression through the traf6-becn1 signaling axis for autophagy induction. Cell Death Dis. (2022) 13:348. doi: 10.1038/s41419-022-04808-7, PMID: 35422093 PMC9010460

[215] Zhan Z Xie X Cao H Zhou X Zhang XD Fan H . Autophagy facilitates tlr4- and tlr3-triggered migration and invasion of lung cancer cells through the promotion of traf6 ubiquitination. Autophagy. (2014) 10:257–68. doi: 10.4161/auto.27162, PMID: 24321786 PMC5396095

[216] Jungnickel C Schmidt LH Bittigkoffer L Wolf L Wolf A Ritzmann F . Il-17c mediates the recruitment of tumor-associated neutrophils and lung tumor growth. Oncogene. (2017) 36:4182–90. doi: 10.1038/onc.2017.28, PMID: 28346430

[217] Dajon M Iribarren K Petitprez F Marmier S Lupo A Gillard M . Toll like receptor 7 expressed by Malignant cells promotes tumor progression and metastasis through the recruitment of myeloid derived suppressor cells. Oncoimmunology. (2019) 8:e1505174. doi: 10.1080/2162402x.2018.1505174, PMID: 30546943 PMC6287801

[218] Liu Z Shan S Yuan Z Wu F Zheng M Wang Y . Mitophagy bridges DNA sensing with metabolic adaption to expand lung cancer stem-like cells. EMBO Rep. (2023) 24:e54006. doi: 10.15252/embr.202154006, PMID: 36416244 PMC9900345

[219] Liu Y Cao X . Characteristics and significance of the pre-metastatic niche. Cancer Cell. (2016) 30:668–81. doi: 10.1016/j.ccell.2016.09.011, PMID: 27846389

[220] Shang C Sun Y Wang Y Shi H Han X Mo Y . Cxcl10 conditions alveolar macrophages within the premetastatic niche to promote metastasis. Cancer Lett. (2022) 537:215667. doi: 10.1016/j.canlet.2022.215667, PMID: 35398531

[221] Theivanthiran B Yarla N Haykal T Nguyen YV Cao L Ferreira M . Tumor-intrinsic nlrp3-hsp70-tlr4 axis drives premetastatic niche development and hyperprogression during anti-pd-1 immunotherapy. Sci Transl Med. (2022) 14:eabq7019. doi: 10.1126/scitranslmed.abq7019, PMID: 36417489 PMC10347419

[222] Liu Y Gu Y Han Y Zhang Q Jiang Z Zhang X . Tumor exosomal rnas promote lung pre-metastatic niche formation by activating alveolar epithelial tlr3 to recruit neutrophils. Cancer Cell. (2016) 30:243–56. doi: 10.1016/j.ccell.2016.06.021, PMID: 27505671

[223] Rolfo C Giovannetti E Martinez P McCue S Naing A . Applications and clinical trial landscape using toll-like receptor agonists to reduce the toll of cancer. NPJ Precis Oncol. (2023) 7:26. doi: 10.1038/s41698-023-00364-1, PMID: 36890302 PMC9995514

[224] Smith DA Conkling P Richards DA Nemunaitis JJ Boyd TE Mita AC . Antitumor activity and safety of combination therapy with the toll-like receptor 9 agonist imo-2055, erlotinib, and bevacizumab in advanced or metastatic non-small cell lung cancer patients who have progressed following chemotherapy. Cancer Immunol Immunother. (2014) 63:787–96. doi: 10.1007/s00262-014-1547-6, PMID: 24770667 PMC11028443

[225] Garon EB Spira AI Johnson M Bazhenova L Leach J Cummings AL . A phase ib open-label, multicenter study of inhaled dv281, a tlr9 agonist, in combination with nivolumab in patients with advanced or metastatic non-small cell lung cancer. Clin Cancer Res. (2021) 27:4566–73. doi: 10.1158/1078-0432.Ccr-21-0263, PMID: 34108179

[226] Otsuka T Nishida S Shibahara T Temizoz B Hamaguchi M Shiroyama T . Cpg odn (K3)-toll-like receptor 9 agonist-induces th1-type immune response and enhances cytotoxic activity in advanced lung cancer patients: A phase I study. BMC Cancer. (2022) 22:744. doi: 10.1186/s12885-022-09818-4, PMID: 35799134 PMC9264631

[227] Hirsh V Paz-Ares L Boyer M Rosell R Middleton G Eberhardt WE . Randomized phase iii trial of paclitaxel/carboplatin with or without pf-3512676 (Toll-like receptor 9 agonist) as first-line treatment for advanced non-small-cell lung cancer. J Clin Oncol. (2011) 29:2667–74. doi: 10.1200/jco.2010.32.8971, PMID: 21632509

[228] Manegold C van Zandwijk N Szczesna A Zatloukal P Au JSK Blasinska-Morawiec M . A phase iii randomized study of gemcitabine and cisplatin with or without pf-3512676 (Tlr9 agonist) as first-line treatment of advanced non-small-cell lung cancer. Ann Oncol. (2012) 23:72–7. doi: 10.1093/annonc/mdr030, PMID: 21464154

[229] Matsumoto M Seya T Kikkawa S Tsuji S Shida K Nomura M . Interferon gamma-producing ability in blood lymphocytes of patients with lung cancer through activation of the innate immune system by bcg cell wall skeleton. Int Immunopharmacol. (2001) 1:1559–69. doi: 10.1016/s1567-5769(01)00071-6, PMID: 11515819

[230] Belani CP Chakraborty BC Modi RI Khamar BM . A randomized trial of tlr-2 agonist cadi-05 targeting desmocollin-3 for advanced non-small-cell lung cancer. Ann Oncol. (2017) 28:298–304. doi: 10.1093/annonc/mdw608, PMID: 27831503

[231] Droemann D Goldmann T Tiedje T Zabel P Dalhoff K Schaaf B . Toll-like receptor 2 expression is decreased on alveolar macrophages in cigarette smokers and copd patients. Respir Res. (2005) 6:68. doi: 10.1186/1465-9921-6-68, PMID: 16004610 PMC1187924

[232] Julian MW Shao G Schlesinger LS Huang Q Cosmar DG Bhatt NY . Nicotine treatment improves toll-like receptor 2 and toll-like receptor 9 responsiveness in active pulmonary sarcoidosis. Chest. (2013) 143:461–70. doi: 10.1378/chest.12-0383, PMID: 22878868

[233] Progatzky F Jha A Wane M Thwaites RS Makris S Shattock RJ . Induction of innate cytokine responses by respiratory mucosal challenge with R848 in zebrafish, mice, and humans. J Allergy Clin Immunol. (2019) 144:342–5.e7. doi: 10.1016/j.jaci.2019.04.003, PMID: 31002833 PMC6602583

[234] Silkoff PE Flavin S Gordon R Loza MJ Sterk PJ Lutter R . Toll-like receptor 3 blockade in rhinovirus-induced experimental asthma exacerbations: A randomized controlled study. J Allergy Clin Immunol. (2018) 141:1220–30. doi: 10.1016/j.jaci.2017.06.027, PMID: 28734844

[235] Krusche J Twardziok M Rehbach K Böck A Tsang MS Schröder PC . Tnf-α-induced protein 3 is a key player in childhood asthma development and environment-mediated protection. J Allergy Clin Immunol. (2019) 144:1684–96.e12. doi: 10.1016/j.jaci.2019.07.029, PMID: 31381928

